# Assessment of Biodegradation Efficiency of Polychlorinated Biphenyls (PCBs) and Petroleum Hydrocarbons (TPH) in Soil Using Three Individual Bacterial Strains and Their Mixed Culture

**DOI:** 10.3390/molecules25030709

**Published:** 2020-02-06

**Authors:** Teresa Steliga, Katarzyna Wojtowicz, Piotr Kapusta, Joanna Brzeszcz

**Affiliations:** 1 Department of Reservoir Fluid Production Technology, Oil and Gas Institute–National Research Institute, ul. Lubicz 25 A, 31-503 Krakow, Poland; katarzyna.wojtowicz@inig.pl; 2 Department of Microbiology, Oil and Gas Institute-National Research Institute, ul. Lubicz 25 A, 31-503 Krakow, Poland; piotr.kapusta@inig.pl (P.K.); joanna.brzeszcz@inig.pl (J.B.)

**Keywords:** bacteria, polychlorinated biphenyls (PCBs), inoculation, biodegradation, total petroleum hydrocarbons (TPH), toxicological tests

## Abstract

Biodegradation is one of the most effective and profitable methods for the elimination of toxic polychlorinated biphenyls (PCBs) and total petroleum hydrocarbons (TPH) from the environment. In this study, aerobic degradation of the mentioned pollutants by bacterial strains *Mycolicibacterium frederiksbergense* IN53, *Rhodococcus erythropolis* IN129, and *Rhodococcus* sp. IN306 and mixed culture M1 developed based on those strains at 1:1:1 ratio was analyzed. The effectiveness of individual strains and of the mixed culture was assessed based on carried out respirometric tests and chromatographic analyses. The *Rhodococcus* sp. IN306 turned out most effective in terms of 18 PCB congeners biodegradation (54.4%). The biodegradation index was decreasing with an increasing number of chlorine atoms in a molecule. Instead, the *Mycolicobacterium frederiksbergense* IN53 was the best TPH degrader (37.2%). In a sterile soil, contaminated with PCBs and TPH, the highest biodegradation effectiveness was obtained using inoculation with mixed culture M1, which allowed to reduce both the PCBs (51.8%) and TPH (34.6%) content. The PCBs and TPH biodegradation capacity of the defined mixed culture M1 was verified *ex*-*situ* with prism method in a non-sterile soil polluted with aged petroleum hydrocarbons (TPH) and spent transformer oil (PCBs). After inoculation with mixed culture M1, the PCBs were reduced during 6 months by 84.5% and TPH by 70.8% as well as soil toxicity was decreased.

## 1. Introduction

Polychlorinated biphenyls (PCBs) are synthetic chlorinated biphenyls, which were manufactured on a large scale for half a century and found various industrial applications, such as components of lubricants, transformer oils, dielectric fluids, and plasticizers. It is usually estimated that more than 1.5 million tons of PCBs have been produced worldwide [1]. These compounds are among the most persistent xenobiotic pollutant classes. They remain in the environment for a long time due to their low reactivity and high chemical stability in various conditions. Because of this, their production and application was banned in 1980 [2,3,4]. However, to a large extent they were dispersed and resulted in vast pollution of all ecosystems [5]. The most alarming adverse PCBs property is their tendency to bioaccumulate in lipid tissues and organic components of the soil and in fat tissues of animals and people [6]. PCBs molecules consist of a biphenyl core containing 1 to 10 chlorine atoms substituted in various positions, as a result of which there are 209 various congeners featuring different degrees of chlorination and substitution. Congeners that have benzene rings in one plane and non-substituted *ortho*-positions are the most persistent and feature the highest toxicity [7,8]. The number of chlorine atoms and their arrangement in PCBs molecule are important factors for aerobic biodegradation by oxidative enzymes. The neighbouring non-chlorinated carbon atoms allow for hydroxylation of the ring and formation of arene oxide intermediate compounds, facilitating aromatic ring splitting. In general, PCB congeners with four or less chlorine atoms in a molecule are easily degraded, and with five or more, are more resistant. PCBs are usually first oxidized by biphenyl dioxygenase (BphA) to produce cis-dihydrodiol semi-products, which are subjected to ring *ortho*- or *meta*-cleavage-producing chlorobenzoates [1,9,10].

Bacteria using biphenyl (BP) as a source of carbon and energy play a crucial role in the PCBs degradation. The most active bacteria capable of PCBs metabolizing and most frequently reported in the literature include: *Achromobacter* sp. [6,11], *Acinetobacter* sp. [12], *Bacillus* sp. [13,14], *Burkholderia* sp. [15] *Janibacter* sp. [16], *Mycobacterium* sp. [17], *Ochrobactrum* sp. [18,19], *Pseudomonas* sp. [20,21,22,23,24,25], and *Rhodococcus* sp. [19,26,27,28,29,30,31,32,33].

The removal of toxic and mutagenic, hydrophobic chlorinated aromatic compounds, mainly PCBs, from the environment is one of most important goals of environmental biotechnology. The cleaning of soil polluted with PCBs using conventional methods such as thermal desorption, the plasma method, combustion methods, dechlorination, or soil washing method are frequently too expensive [34]. Biological methods are an alternative strategy for PCBs removal and now continuous development of bioremediation techniques is observed [19,31,35,36,37,38]. Bioremediation is considered an effective and profitable method for PCBs removal from the soil and its main strategies comprise bioaugmentation and/or biostimulation, which means the introduction of bacterial strains, degrading PCBs individually or as a mixed culture, and nutrients, respectively. In order to avoid antagonistic interaction with foreign natural soil microbiota on foreign microbial cultures not adapted to a given environment from a commercial mixed culture, it is preferable to prepare bacterial consortia based on previously isolated, selected and multiplied indigenous microorganisms. They do not upset the biological equilibrium, creating a high probability of survival in polluted biocenosis, and hence guarantee an appropriately high biodegradation potential [39,40,41,42]. The bioaugmentation may fail due to the following reasons: cell stress, lack of nutrients, existence of more preferred source of carbon as a substrate, competition, or inappropriate biological availability. The studies prove that the application of mixed bacterial culture better reflects real conditions as compared with the application of a pure bacterial culture [43]. There is little information in the literature about the application of bacterial consortia to clean soils historically contaminated by PCBs. Havel and Reineke [44] applied a consortium containing isolates belonging to *Pseudomonas* sp., and Egorova et al. [45] used a combination of strains *Rhodococcus ruber* P52 and *Microbacterium sp* B51. The last study carried out by Horváthová et al., [19] showed clear degradation of highly chlorinated PCB congeners by means of bacterial consortia prepared on the basis of four bacterial strains (*Achromobacter xylosoxidans, Stenotrophomonas maltophilia, Ochrobactrum anthropi and Rhodococcus ruber*) at various biomass ratios, isolated from deposits historically contaminated with PCBs. Satisfactory effects of PCBs biodegradation (60% after 8 weeks) were achieved using as an inoculum of a mixture of four bacterial strains: *Bacillus* sp., *Achromobacter* sp., *Pseudomonas stutzeri*, and *Bacillus subtilis* [38].

Apart from biological factors, the effectiveness of PCBs group biodegradation is affected by environmental factors, such as temperature, pH, oxygen availability, humidity, soil matrix properties, nutrients content [46], a surfactant addition [47], as well as pollution-related factors (physical and chemical properties of polychlorinated biphenyls, xenobiotic concentration, pollutant age, number of chlorine atoms in a biphenyl ring, and a spatial PCBs structure) [48].

Frequent historical soil contamination with polychlorinated biphenyls (PCBs) originating from a transformer oil is accompanied by the existence of total petroleum hydrocarbons (TPH) [31]. Biodegradation of petroleum pollutants in a historically polluted soil originating from waste pits with the use of a defined mixed culture based on non-pathogenic indigenous bacterial strains was studied by the authors, which is proven by numerous papers [39,49,50,51].

The studies were aimed at the determination of aerobic biodegradation effectiveness PCBs and TPH with the use of three single bacterial strains isolated from contaminated natural environments and a mixed culture developed on the basis of these strains at 1:1:1 ratio. The studies were carried out on a sterile soil polluted with (a) PCBs, (b) TPH, and (c) both PCBs and TPH. The effectiveness of individual strains and of the mixed culture was assessed based on carried out respirometric tests and chromatographic analyses of PCBs and TPH in the examined soil. The PCBs and TPH biodegradation capacity of the defined mixed culture was verified under semi-technical conditions by means of the ex-situ prism method on a non-sterile soil weathered polluted with petroleum hydrocarbons (TPH) and spent PCBs containing transformer oil. The course of PCBs and TPH biodegradation in the soil was monitored using chromatographic determination of the content of individual polychlorinated biphenyls and petroleum hydrocarbons as well as toxicological studies using a set of new-generation toxicological tests, in which the bioindicators belong to various taxonomic groups (bacteria, crustaceans, and higher plants).

## 2. Results

### 2.1. Bacterial Strains Used in this Study

The 16S rRNA gene sequences of strains used in this study have been deposited in the NCBI GenBank database and are available under the following accession numbers: JN572675 (*Mycolicibacterium frederiksbergense* IN53), KT923311 (*Rhodococcus erythropolis* IN129), and KX058399 (*Rhodococcus* sp. IN306). Table 1 presents the similarity of our strains with their closest relatives based on the available 16S rRNA gene sequences deposited in the NCBI GenBank database (comparison was made both for individual nucleotide sequences and the whole genomes). For the genomes, annotated genes coding putative enzymes responsible for degradation of polycholorinated biphenyls and other hydrocarbons, are shown. The presented data show that genomes of organisms closely related to strains IN53, IN129, and IN306 contain genes responsible for the catabolism of both aliphatic and aromatic hydrocarbons. All genome sequences contain genes coding for the third enzyme of the biphenyl degradation pathway (Table 1), i.e., 2,3-dihydroxybiphenyl 1,2-dioxygenase (*bphC*). Moreover, each genome contains more than one copy of this gene (Table 1). In the genome sequence of *Rhodococcus jostii* RHA1, all genes are related to the enzyme responsible for the initial stages of biphenyl degradation. Namely, genes encoding for biphenyl 2,3-dioxygenase (*bphA*, found all four components), biphenyl-2,3-dihydrodiol 2,3-dehydrogenase (*bphB*), and 2,3-dihydroxybiphenyl 1,2-dioxygenase (*bphC*) (Table 1). The genome sequence of *Mycobacterium* sp. YC-RL4 contains a gene coding for the sub-unit alpha of ring-hydroxylating dioxygenase (probably the first component of biphenyl 2,3-dioxygenase, Table 1), and there are genes coding the same enzyme and the enzyme identified as the biphenyl 2,3-dioxygenase in the genome of *Rhodococcus erythropolis* X5. In addition, all the analyzed genome sequences contain *alkB*, the gene coding for alkane 1-monooxygenase. This enzyme is responsible for the hydroxylation, which is the first reaction in the alkane degradation pathway.

**Figure 1:**
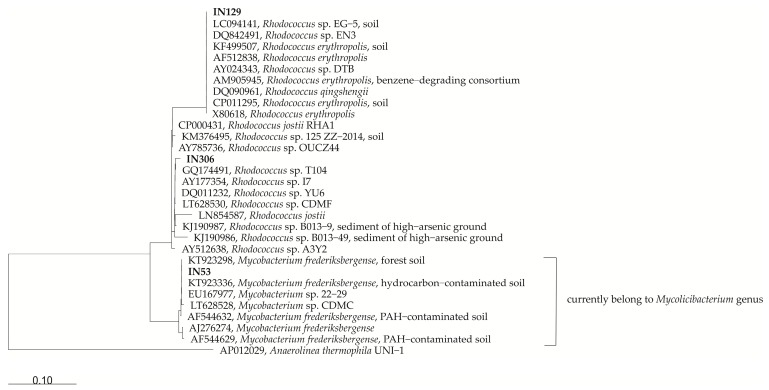
Phylogenetic consensus tree of the stains IN129, IN53 and IN306 with closely related sequences from the NCBI GenBank database. GenBank accession numbers are shown in the Figure. The scale bar corresponds to 10% estimated sequence divergence.

For the tree construction, the 16S rRNA gene sequences of the IN53, IN129 and IN306 were aligned using SINA Aligner, [52] and further analyzed using ARB program [53] with the SILVA SSU Ref NR99 release 132 database. Phylogenetic reconstructions were performed with full-length sequences using Neighbour Joining with Jukes-Cantor correction and Maximum Likelihood (RaxML, model: GTRGAMMA). In all cases, a base frequency filter was applied. The selected tree represents a consensus topology between the different reconstructions (Figure 1).

### 2.2. Assessment of PCB and TPH Biodegradation Effectiveness in Sterile Soils

#### 2.2.1. Respirometric Tests

An increase in the microbiological activity in a reactive environment proves that microorganisms used PCBs and TPH as a source of carbon and energy. An increased biological activity in the system results in a growth of oxygen consumption by the studied bacterial strains: *Mycolicibacterium frederiksbergense* IN53, *Rhodococcus erythropolis* IN129, *Rhodococcus* sp. IN306, and mixed culture M1 (prepared from individual bacterial strains at 1:1:1 ratio), over time causing biodegradation of analytes.

**Figure 2:**
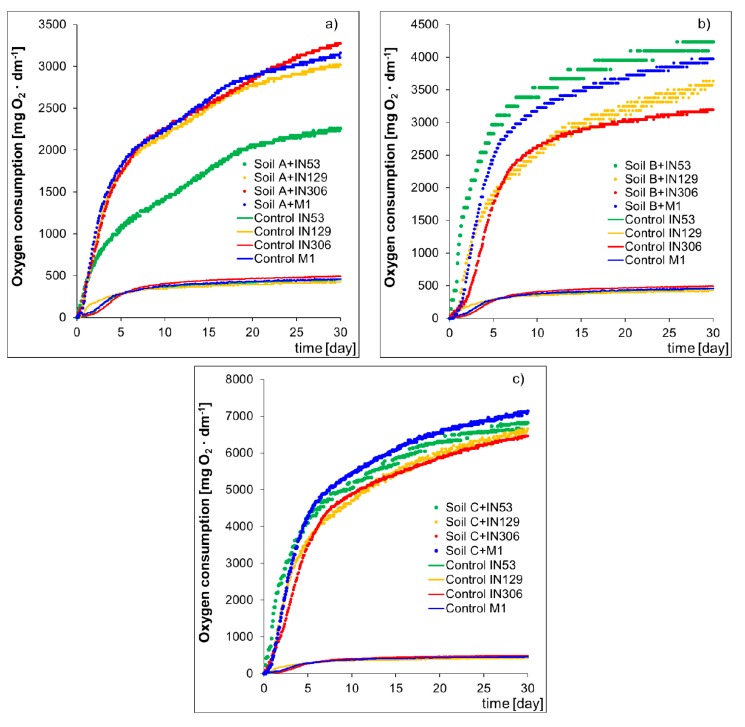
Oxygen consumption by microorganisms Mycolicibacterium frederiksbergense IN53, Rhodococcus erythropolis IN129, Rhodococcus sp. IN306 and mixed culture M1 to degrade (**a**) PCBs (sterile Soil A contaminated PCB), (**b**) TPH (sterile Soil B contaminated TPH), and (**c**) PCBs and TPH (sterile Soil C contaminated PCB and TPH) in the biodegradation process. Control-sterile uncontaminated “pure” soil inoculated with bacterial strains and mixed culture.

Figure 2 presents the dependence of the consumed oxygen amount on the experiment duration for samples of sterile Soils A, B, and C inoculated with strains of bacteria IN53, IN129, IN306, and mixed culture M1 for control samples. The respirometric tests performed on a sterile soil contaminated with PCBs (Soil A) inoculated with strains IN53, IN129, IN306 and mixed culture M1 showed that the process of biodegradation is most intensive during inoculation with *Rhodococcus* sp. IN306, which is proved by the amount of consumed oxygen after 30 days of testing, approx. 3,274 ± 196 mg O_2_/dm^3^. A slightly lower oxygen consumption during the experiment was recorded in the sample inoculated with mixed culture M1 (3,161 ± 174 mg O_2_/dm^3^) and with *Rhodococcus erythropolis* IN129 (3,020 ± 172 mg O_2_/dm^3^). *Mycolicibacterium frederiksbergense* IN53 turned out to be least effective in terms of biodegradation potential of PCBs group compounds, because the oxygen consumption after 30 days of testing amounted to 2258 ± 124 mg O_2_/dm^3^. The results of respirometric tests performed on the sample of Soil B showed that the strain IN53 features the highest biodegradation potential in terms of TPH content reduction in the soil (4235 ± 241 mg O_2_/dm^3^). Slightly lower amounts of consumed oxygen after the test completion were recorded for samples inoculated with mixed culture M1 (3975 ± 226 mg O_2_/dm^3^) and with the IN129 strain (3636 ± 204 mg O_2_/dm^3^). The lowest biological activity after completion of the respirometric test was observed in the sample inoculated with the IN306 strain (3193 ± 179 mg O_2_/dm^3^). Results of respirometric tests carried out on the sample of soil contaminated both with PCBs and TPH (Soil C) showed that the biodegradation is most intensive in the sample inoculated with mixed culture M1 (7136 ± 357 mg O_2_/dm^3^). Inoculation of Soil C with strains IN53, IN129, and IN306 also resulted in an increase in the microbiological activity in the system, which is proved by the amounts of consumed oxygen after 30 days of the experiment, 6817 ± 348 mg O_2_/dm^3^, 6656 ± 339 mg O_2_/dm^3^, and 6466 ± 336 mg O_2_/dm^3^, respectively.

#### 2.2.2. Assessment of PCBs and TPH Biodegradation Based on Chromatographic Analyses

**Figure 3:**
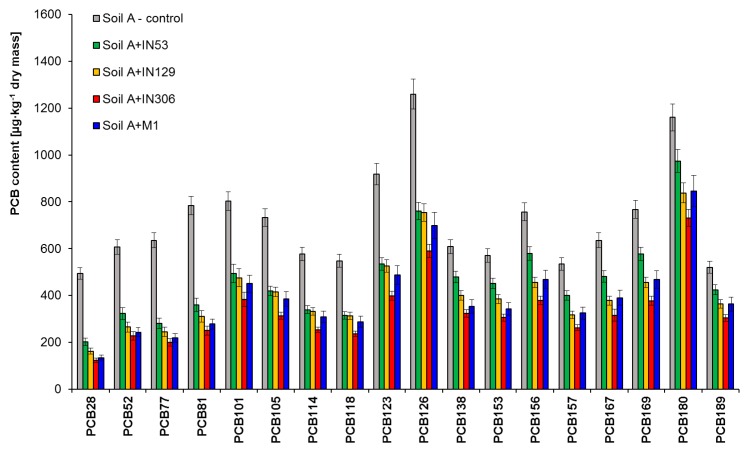
Residual PCB congeners content in sterile Soil A contaminated with PCBs inoculated by Mycolicibacterium frederiksbergense IN53, Rhodococcus erythropolis IN129, Rhodococcus sp. IN306 and mixed culture M1 after a 30-day biodegradation process, (repetition number *n* = 8–10, *p* < 0.05). Control-non-inoculated Soil A.

PCB biodegradation potential of studied bacterial strains and mixed culture in sterile soil contaminated solely with PCBs (Soil A) was assessed by chromatographic analyses. Figure 3 and Table S1 present the content of studied PCB congeners in the PCBs contaminated soil (Soil A) before and after 30-day inoculation with bacterial strains: *Mycolicibacterium frederiksbergense* IN53, *Rhodococcus erythropolis* IN129, *Rhodococcus* sp. IN306, and mixed culture M1.

The results of chromatographic analyses showed that *Rhodococcus* sp. IN306 features the highest biodegradation potential with respect to the studied PCB congeners. The inoculation of soil A with the strain IN306 led to a significant reduction of the PCB content from 13,100 to 5,970 µg/kg of dry mass (54.4%). Satisfactory results were achieved also for mixed culture M1 from 13,100 µg/kg to 6309 µg/kg of dry mass (46.2%). *Mycolicibacterium frederiksbergense* IN53 turned out to be the least effective, for which the degree of biodegradation after 30 days of test duration was 35.9%. The PCB28 congener (containing three chlorine atoms in a molecule) turned out to be the most easily biodegradable, in which content in the sample after 30 days of testing was reduced from 494 to 202 µg/kg of dry mass, (IN53), 162 µg/kg (IN129), 123 (IN306), and 134 (M1), which is 59.1%, 67.2%, 75.1%, and 72.9%, respectively. Congeners containing four chlorine atoms in a biphenyl molecule, including: PCB52, PCB77, and PCB81 were also degraded to a comparable extent: PCB52 (46.8–62.4%), PCB77 (55.9–68.5%), and PCB81 (54.1–68.1%). PentaCBs (PCB101, PCB105, PCB114, PCB118, PCB123, and PCB126) were reduced as follows: 38.5–42.8% (IN53), 40.8–43.4% (IN129), 52.3–57.2% (IN306), and 43.9–47.4% (M1). The PCB105 congener turned out to be the most easily biodegradable among PentaCBs, in which thecontent in the sample was reduced from 733 µg/kg of dry mass to 419 µg/kg (IN53), 414 µg/kg (IN129), 313 µg/kg (IN306), and 385 µg/kg of dry mass (M1). The PCB congener containing five chlorine atoms in a molecule most difficult to degrade was PCB101 (di-*ortho* structure), because the degree of its biodegradation as a result of inoculation was: 38.5% (IN53), 40.1% (IN129), 52.3% (IN306), and 49.8% (M1). The HexaCBs content reduction ranged from 23.4 to 49.3%. Among HexaPCBs, the PCB157 inoculated with *Rhodococcus* sp. (IN306), showed the highest reduction of content after 30 days of testing—by 51.2%, while the most difficult to degrade were: PCB138 and PCB153, for which the biodegradation degrees were 46.9% and 46.5%, respectively. HeptaCBs (PCB180 and PCB189) turned out to be most difficult to degrade, for which the highest biodegradation degree after inoculation with IN306 was 31.7% and 38.7%.

**Figure 4:**
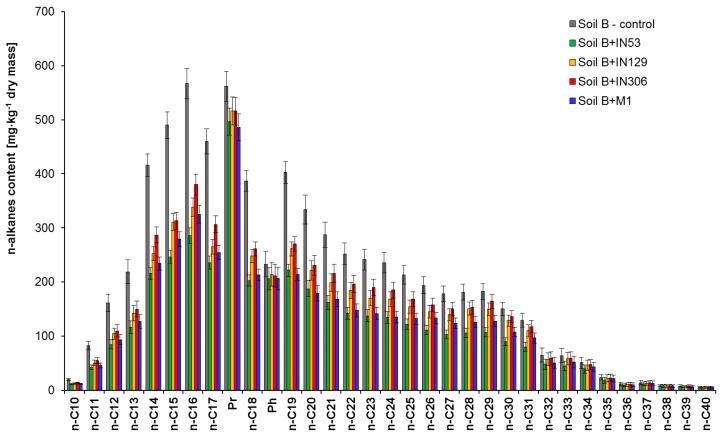
Residual n-alkanes content in sterile Soil B contaminated with TPH inoculated by Mycolicibacterium frederiksbergense IN53, Rhodococcus erythropolis IN129, Rhodococcus sp. IN306 and mixed culture M1 after a 30-day biodegradation process, (repetition number *n* = 8–10, *p* < 0.05). Control non-inoculated Soil B.

Chromatographic analyses were carried out on sterile soil contaminated solely with TPH (Soil B) before and after 30-day inoculation with: *Mycolicibacterium frederiksbergense* IN53, *Rhodococcus erythropolis* IN129, *Rhodococcus* sp. IN306, and mixed culture M1, which showed that the strain *Mycolicibacterium frederiksbergense* IN53 features the highest biodegradation potential with respect to TPH. Table S2 and Figure 4 present a comparison of the content reduction for TPH; unidentified hydrocarbons; alkanes; ΣnC_10_–nC_22_, ΣnC_23_–nC_40_; and individual n-alkanes in Soil B after inoculation with strains IN53, IN129, IN306, and mixed culture M1.

The TPH content in Soil B after 30 days of Soil B inoculation with the *Mycolicibacterium frederiksbergense* IN53 was reduced by 37.2% (from 12,515 to 7863 mg/kg of dry mass). A satisfactory reduction of TPH content from 12,515 to 8309 mg/kg of dry mass (33.6%) was recorded during inoculation with mixed culture M1. The TPH content in Soil B samples inoculated with IN129 and IN306 strains was reduced by 28.5% and 24.9%, respectively. The easiest to degrade were aliphatic hydrocarbons with chain lengths nC_10_–nC_22_, within a range of: 42.7–48.7% (IN53), 38.7–41.5% (IN129), 24.4–33.4% (IN306), and 39.5–46.7% (M1). In turn, heavier homologues (nC_23_–nC_40_) were degraded less efficiently and their content was reduced by 21.0% (IN129), 15.2% (IN306), and 31.8% (M1) in samples inoculated with IN129, IN306 and M1, respectively. The best effects of heavy hydrocarbons biodegradation were obtained during Soil B inoculation with the *Mycolicibacterium frederiksbergense* IN53 strain, equal to 39.5%.

A satisfactory effect of n-alkanes biodegradation as a result of Soil B inoculation is proven by indices of C_17_/Pr and C_18_/Ph biodegradation, which were substantially reduced (Table S2). The highest reduction was recorded during inoculation with the IN53 strain (C_17_/Pr from 0.818 to 0.473 and C_18_/Ph from 1.675 to 0.985) and with mixed culture M1 (C_17_/Pr from 0.818 to 0.522 and C_18_/Ph from 1.675 to 1.029).

**Figure 5:**
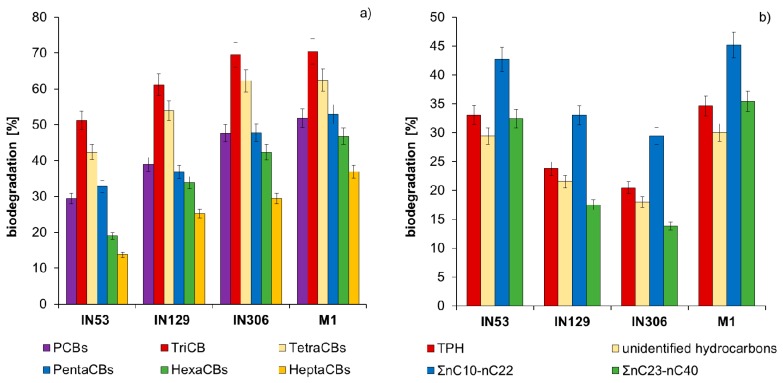
Comparison of biodegradation (%) in sterile Soil C contaminated with both PCBs and TPH after inoculation with Mycolicibacterium frederiksbergense IN53, Rhodococcus erythropolis IN129, Rhodococcus sp. IN306, and mixed culture M1: (**a**) polychlorinated biphenyls; (**b**) petroleum hydrocarbons (repetition number *n* = 8–10, *p* < 0.05).

The comparison of PCBs and TPH content in sterile Soil C after inoculation with strains IN53, IN129, IN306, and mixed culture M1 is presented in Tables S1 and S2 and in Figure 5. Results of chromatographic analyses proved that during the inoculation of sterile Soil C with mixed culture M1 a high reduction of PCBs content was achieved from 13,100.0 to 6309 µg/kg of dry mass (51.8%) as well as of TPH content from 12,365 to 8085 mg/kg of dry mass (34.6%). The TriCBs content after 30 days of inoculation was reduced by 51.2% (*Mycolicibacterium frederiksbergense* IN53), 61.1% (*Rhodococcus erythropolis* N129), 69.4% (*Rhodococcus* sp. IN306), and 70.4% (M1). TetraCBs were biodegraded to a comparable extent: 43.3% (IN53), 53.9% (IN129), 62.3% (IN306), and 62.4% (M1). PentaCBs and HexaCBs were best degraded by the mixed culture M1, by 52.9% and 46.8%. HeptaCBs were definitely the most difficult to biodegrade among PCBs. Their content as a result of inoculation with strains IN53, IN129, IN306, and mixed culture M1 was reduced from 1680 µg/kg of dry mass to: 1449 µg/kg (13.8%), 1256 µg/kg (25.2%), 1184 µg/kg (29.5%), and 1059 µg/kg (37.0%), respectively.

Chromatographic analyses of petroleum hydrocarbons (TPH) showed that aliphatic hydrocarbons nC_10_–nC_22_ were easiest to degrade, for which the biodegradation degree after 30 days of inoculation ranged as follows: 41.1–48.2% (IN53), 35.3–39.1% (IN129), 26.2–34.2% (IN306), and 39.1–45.4% (M1). Long chain aliphatic hydrocarbons nC_23_–nC_40_ were reduced by 32.5% (IN53), 17.5% (IN129), 13.8% (IN306), and 35.4% (M1) accordingly. Biodegradation of unidentified hydrocarbons reached: 29.4% (IN53), 21.5% (IN129), 18.0% (IN306), and 30.0% (M1).

### 2.3. Assessment of PCBs and TPH Biodegradation in Non-Sterile Soil-the Ex-Situ Prism Method

The effectiveness of mixed culture M1 was verified carrying out the process of biodegradation in a non-sterile Soil D collected from waste pit G-44 with a TPH content of 22,126 mg/kg of dry mass, which was contaminated with aged transformer oil containing PCBs up to 8752 µg/kg of dry mass. Figure S1 illustrates the percentage share of identified pollutants in the sample (Soil D) comprising: (a) PCBs, (b) TPH.

**Figure 6:**
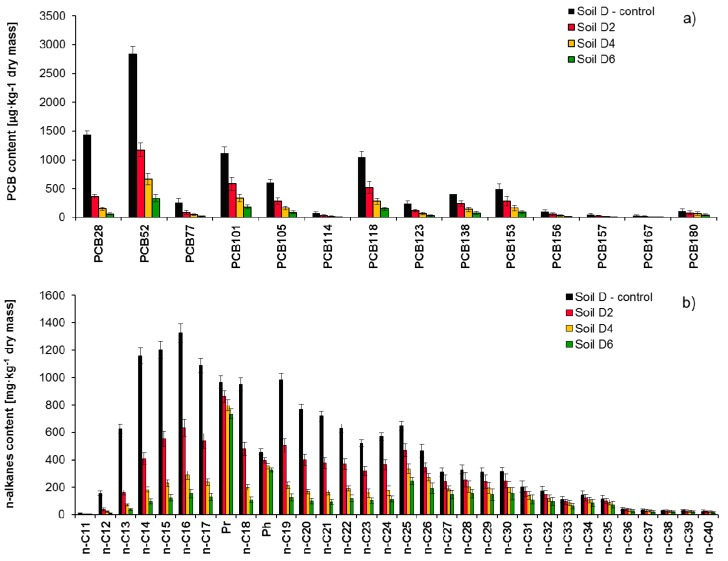
Residual content of (**a**) PCB congeners and (**b**) n-alkanes in non-sterile soil contaminated with both PCBs and petroleum hydrocarbons (Soil D) after 2 (Soil D2), 4 (Soils D4) and 6 (Soil D6) months of inoculation with mixed culture M1 (ex-situ prism method), (repetition number *n* = 7–10, *p* < 0.05). Control non-inoculated Soil D.

Comparison of identified pollutants content in the studied soil (Soil D) and after 2 (Soil D2), 4 (Soil D4), and 6 (Soil D6) months of inoculation with mixed culture M1 is presented in Figure 6: (a) PCB congeners, (b) n-alkanes. As a result of Soil D inoculation with mixed culture M1 after 6 months of the process duration, the content of identified PCBs was reduced from 8752 µg/kg of dry mass to 1357 µg/kg of dry mass (84.5%). Results of Soil D2 chromatographic analyses show that the PCB28 content was reduced from 1396 µg/kg to 371 µg/kg of dry mass (73.4%). The tetraCBs content (PCB52 and PCB77) decreased by 56.4 and 65.6% respectively. The biodegradation of pentaCBs was similar and ranged from 49.8 to 53.6%. In this case, the *di*-*ortho* PCB101 was an exception, in which biodegradation degree was much lower and after 2 months of the process amounted to 45.2%. *Di*-*ortho* congeners turned out to be most durable among hexaCBs, in which content decreased by 40.1% (PCB138) and 39.3% (PCB156). The other congeners having six chlorine atoms were reduced by 44.8 to 45.3%. The PCB180 congener turned out to definitely be the most difficult to degrade, which has seven chlorine atoms and a *di*-*ortho* spatial structure (29.7%).

After 4 months (Soil D4), the content of low-chlorinated biphenyls: PCB28, PCB52, and PCB77 decreased by 89.7%, 76.1%, and 81.2%, respectively. PCB101 was reduced by 69.5%, while the other PentaCBs between 72.1 and 73.3%. The HexaCBs (PCB 138, PCB 153, PCB 156, and PCB 167) content reduction ranged from 65.9 to 69.8%, while the PCB180 biodegradation in soil D4 was 40.8%. The process of Soil D inoculation with mixed culture M1 was finished after 6 months, and the recorded reduction of individual identified congeners was: PCB28, PCB52, and PCB77 (87.7–94.6%); pentaCBs (83.3–86.7%); HexaCBs (81.0–84.2%); and PCB180 (58.6%). The inoculation of non-sterile Soil D with mixed culture M1 during 6 months resulted in a TPH content reduction from 22,126 to 6470 mg/kg of dry mass. The degree of TPH content reduction during the process of inoculation with mixed culture M1 was as follows: after 2 months, 41.4%; after 4 months, 60.7%; and after 6 months, 70.8%.

The chromatographic analysis showed that during inoculation with mixed culture M1, the biodegradation of aliphatic hydrocarbons nC_11_–nC_22_ was as follows: Soil D2 (47.2–76.2%), Soil D4 (77.5–88.7%), and Soil D6 (87.2–93.2%). Hydrocarbons from the nC_23_–nC_40_ range were also degraded to a satisfactory degree: Soil D2 (13.8–41.2%), Soil D4 (20.1–62.9%), and Soil D6 (25.6–72.4%). The content of unidentified hydrocarbons in Soil D6 was reduced by 68.1%. The assessment indices of biodegradation degree proved a high level of biological decomposition of n-alkanes, because they were visibly reduced in Soil D6: nC_17_/Pr from 1.131 to 0.192, nC_18_/Ph from 2.081 to 0.402.

**Figure 7:**
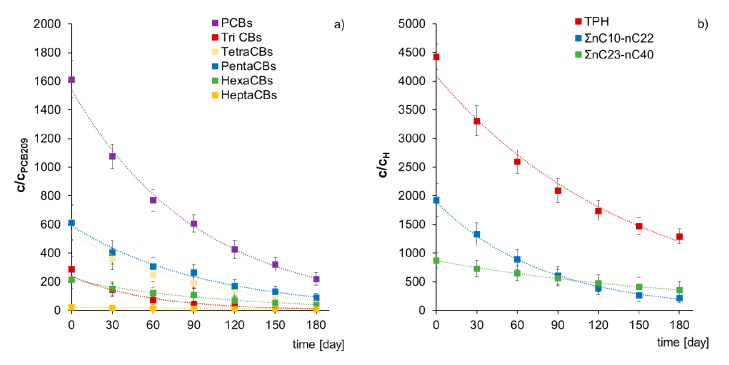
Comparison of content reduction (**a**) in PCB-209 normalized PCBs, TriCB, TetraCBs, PentaCBs, HexaCBs, and HeptaCBs (**b**) in hopane-normalized TPH, ΣnC_10_–nC_22_, and ΣnC_23_–nC_40_ during inoculation of non-sterile soil D with mixed culture M1 (ex-situ prism method). (repetition number *n* = 7–10, *p* < 0.05).

The course of PCBs and TPH biodegradation process during the carried out inoculation with mixed culture M1 of non-sterile Soil D is described by equation (2). Individual coefficients of equation (2): k, (C/C_x_)_0_, and the correlation coefficient (r^2^) are specified in Table S3. A comparison of the biodegradation course is presented in a graphical form for PCBs, TriCBs, TetraCBs, PentaCBs, HexaCBs, HeptaCBs (Figure 7a), and for TPH, ΣnC_10_–nC_22_, and ΣnC_23_–nC_40_ (Figure 7b).

The first-order biodegradation constants (k) during inoculation of non-sterile Soil D with mixed culture M1 are as follows: PCBs (0.0109 d^−1^), TriPCB (0.0170 d^−1^), TetraCBs (0.0110 d^−1^), PentaCBs (0.0095 d^−1^), Hexa CBs (0.0082 d^−1^), and HeptaCBs (0.0050 d^−1^). Instead, for petroleum pollutants, they are: TPH (0.0070 d^−1^), Σ nC_11_–nC_22_ (0.0130 d^−1^), and Σ nC_23_–nC_40_ (0.0049 d^−1^). The presented results allow comparing the kinetics of individual pollutant groups biodegradation during inoculation with mixed culture M1 and prove its biodegradation capacity both of contamination with polychlorinated biphenyls (PCBs) and total petroleum hydrocarbons (TPH).

#### Ecotoxicological Assessment

**Figure 8:**
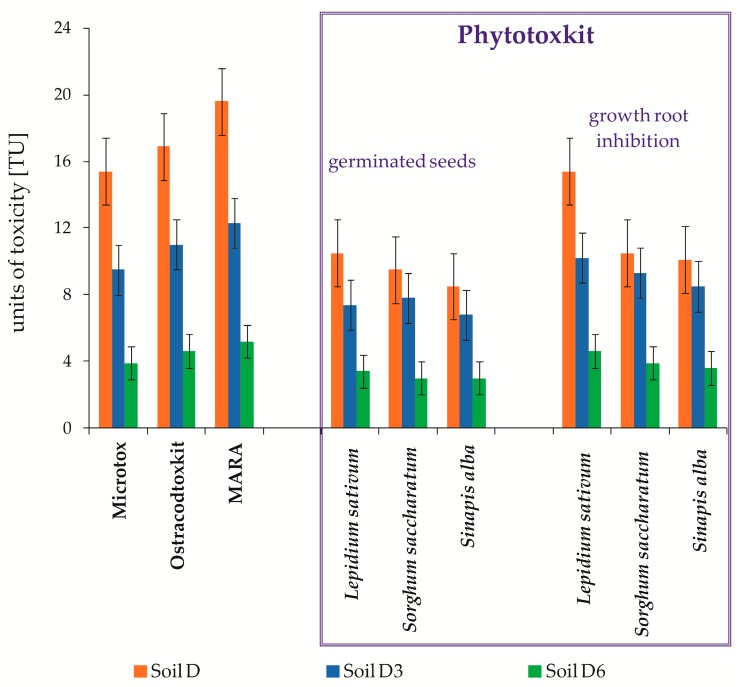
Comparison of toxicity test results expressed in toxicity units (TU) from non-sterile soil during inoculation with mixed culture M1 (ex-situ prism method) (*n* = 3, *p* < 0.05): Soil D—raw soil; Soil D3—soil after 3 months; Soil D6—soil after 6 months.

During petroleum hydrocarbons biodegradation, metabolites of diversified or poorly recognized biological activity can originate as a result of chemical and microbiological changes. Because of that, it is recommended to carry out toxicological tests, which enable monitoring the toxicity of PCBs and TPH biodegradation during inoculation with mixed culture M1 in a non-sterile soil (Soil D) under semi-technical conditions by the ex-situ prism method. Toxicological tests on the trophic level of reducers were carried out by means of Microtox SPT test using luminescent bacteria *Vibrio fischeri*. The determined EC_50_ concentration causing 50% inhibition of test bacteria luminescence was 6.5% vol., which corresponds to a toxicity expressed in toxicity units of around TU = 15.4. Toxicological analyses carried out during Soil D inoculation with mixed culture M1 showed a decrease of its toxicity during 6 months to a level of TU = 3.9, at which the cleaned soil may be classified as low-toxic (Figure 8).

The next test, which was applied to estimate Soil D toxicity on the consumer level during inoculation with mixed culture M1, was the Ostracodtox kit based on the use of *Heterocypris incongruens* organisms, which are more sensitive to pollutants than the *Vibrio fisheri* bacteria. Figure 8 presents the test results. The toxicity (TU) of raw soil (Soil D) was 16.9 and was gradually going down to TU = 11.3 (after 3 months) and TU = 4.9 (after 6 months).

Moreover, an innovative MARA test was performed, estimating the environmental risk and using 11 test strains, which featured various sensitivities. The toxicity calculated based on the mean toxic concentration MCT mean, expressed in toxicity units, was 19.6. During the carried out PCBs and TPH biodegradation, its reduction to 5.2 was recorded (Figure 8).

In the toxicological tests on the producers level, the Phytotoxkit test was used, in which the tested plants were: *Lepidium sativum, Sorghum saccharatum*, and *Sinapis alba*. After the conversion of toxic concentrations to toxicity units (TU), considering the plant germination as a criterion of toxicity assessment, it was found that during the carried out biodegradation process of pollutants contained in the tested soil samples, it was gradually decreasing from 10.5 to 3.0 (*Lepidium sativum*), from 9.5 to 3.1 (*Sorghum saccharatum*), and from 8.5 to 3.0 (*Sinapis alba*). For the second tested parameter (root growth inhibition), the calculated TU value for the tested plants in the sample (Soil D) ranged from 10.1 to 15, while after inoculation with mixed culture M1 (Soil D6), it went down to a level of 3.6–4.6. The carried out tests showed that *Lepidium sativum* was the plant most sensitive to pollutants contained in the tested soil samples (Figure 8).

**Figure 9:**
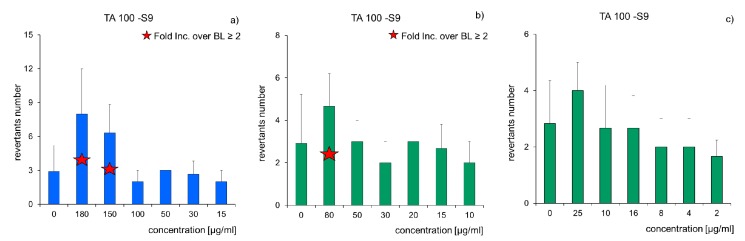
Influence of PCBs and TPH concentration on revertants number (*n* = 3, *p* < 0.05): (**a**) raw soil (Soil D); (**b**) soil after 3-month biodegradation (Soil D3); (**c**) soil after 6-month biodegradation (Soil D6); fold increase over the baseline (mutagenicity index).

The Ames test series was applied to find mutagenic and carcinogenic compounds in raw and cleaned soil. A standard strain TA-100 (*Salmonella typhimirium*) was used. Its mutations exist on a histidine operon, which is not capable of amino acid synthesising. It was observed that the application of an activator (microsomal S9 fraction) had an insignificant impact on the growth of reversible mutations number. Results of tests on raw samples and in the consecutive months of soil bioremediation are presented in Figure 9 as changes of revertants number versus the pollutant concentrations. In the case of (Soil D) samples, which are considered mutagenic, the mutagenecity coefficient ranged from 7.85 to 9.85 (Figure 9a). During the carried out process of pollutants (PCBs and TPH) biodegradation, the mutagenecity coefficient was gradually decreasing: Soil D3 from 4.32 to 6.19 (Figure 9b), Soil D6 from 0.68 to 1.86 (Figure 9c), i.e., to a level at which the number of revertants induced without histidine was slightly higher (less than twice) than the number of spontaneous mutants in the control sample. Therefore, the samples cannot be classified as mutagenic.

## 3. Discussion

Polychlorinated biphenyls (PCBs), despite chemical stability and toxicity, can be degraded under aerobic conditions or at least reduced to low-chlorinated congeners by microorganisms possessing genes encoding for biphenyl dioxygenase responsible for the onset of a specific metabolic degradation pathway [3,54]. The literature studies prove that the best PCBs biodegradation properties are featured by bacteria isolated from the soil/deposits historically PCBs contaminated: *Microbacterium oleivorans, Stenotrophomonas maltophilia*, *Brevibacterium* sp., *Ochrobactrum anthropi, Pseudomonas mandelii, Rhodococcus* sp., *Achromobacter xylosoxidans, Stenotrophomonas* sp., *Ochrobactrum* sp. [6], *Sinorhizobium meliloti* [35,55], *Rhodococcus ruber* and *Rhodococcus pyridinivoran* [33], *Rhodococcus* sp. and *Stenotrophomonas maltofilia* [56] *Bacillus* sp., *Achromobacter* sp., *Pseudomonas stutzeri*, [38]. Results of genes sequencing for bacterial strains isolated from the deposit polluted with transformer oil showed that *Rhodococcus* sp., *Pseudomonas* sp., *Pseudoxanthomonas* sp., *Agromyces* sp., and *Brevibacillus* sp. were the prevailing bacteria decomposing PCBs [57]. *Rhodococcus jostii* RHA1 is one of the best characterized PCBs degraders, featuring a broad PCBs degradation range and its catabolic potential and adaptation to the soil environment suggest that it can be appropriate for bioremediation of polluted Soil D [31].

Research conducted to determine the biodegradability of PCB congeners in sterile contaminated (i.e., one that does not contain other living organisms) reference soil PCB SQC068 by single bacterial strains isolated from contaminated natural environments (*Mycolicibacterium frederiksbergense* IN53, *Rhodococcus erythropolis* IN129, *Rhodococcus* sp. IN306) and the mixed culture M1 prepared on the basis of these strains at ratio 1:1:1, confirm the anticipated ability of these strains to metabolize biphenyl and its derivatives. The mixed culture M1 confirms the anticipated ability of these strains to metabolize biphenyl and its derivatives. It was possible to show that each of the tested bacterial stains, as well as their mixture, degraded PCB congeners, where the *Rhodococcus* sp. IN306 strain turned out to be most effective, removing more than 54.4% of PCBs. *Rhodococcus* sp. IN306 strain is very closely related to the *Rhodococcus jostii* RHA-1 species (Figure 1, Table 1), which is a well-known degrader of biphenyl and its polychlorinated derivatives [31,58,59,60]. The other two strains featured a lower degree of degradation, but the obtained results confirm the observations made during previous metabolic tests, that they are capable of carrying out catabolic processes of biphenyl and its derivatives. In the case of *Rhodococcus erythropolis* IN129 [26,29], it is not surprising, because other species belonging to the *Rhodococcus genus* [27,30,32,33,61,62] have such abilities; in the case of *Mycolicibacterium frederiksbergense* IN53, this means that this strain has even more potential for degrading xenobiotics than previously thought. The higher biodegradation efficiency of polychlorinated biphenyls in the case of *Rhodococcus* sp. IN306 compared to the mixed culture M1 most likely results from competition between individual strains for the same substrates.

Moreover, the obtained results of chromatographic analyses show that with an increasing number of chlorine atoms in a PCB molecule, the degree of their degradation decreases (Table S1), which is consistent with other studies [2,9,54]. PCB congeners containing up to four chlorine atoms in a molecule easily decompose as a result of carried out inoculation with all tested bacterial strains and mixed culture M1 developed based on these strains. Highly chlorinated biphenyls (more than four chlorine atoms in a molecule) are definitely more difficult to degrade [63], in the case of which satisfactory results were obtained for samples inoculated with *Rhodococcus* sp. IN306 and mixed culture M1. For example, the content of PCB-28 congener with three chlorine atoms was reduced as a result of *Rhodococcus* sp. IN306 action by 75.1%, while the content of pentaCBs, hexaCBs, and heptaCBs was reduced by 55.3%, 49.2%, and 39.3%, respectively. It should be noticed that in the case of polychlorinated biphenyls biodegradation, their spatial structure is also important, apart from the number of chlorine atoms in a molecule [64]. It was observed that so-called *di*-*ortho* congeners (two chlorine atoms in an -*ortho* position) are more difficult to biodegrade as compared with *non*-*ortho* or *mono*-*ortho* PCBs with the same number of chlorine atoms in the biphenyl ring [9,65]. 5 *di*-*ortho* PCB congeners (PCB52, PCB101, PCB138, PCB153, and PCB180) were determined in the studied sample (Soil A). For example, as a result of standard PCB Soil A inoculation with the *Rhodococcus* sp. IN306 strain, the reduction of *di*-*ortho* PCB52 congener (tetrachlorobiphenyl) was 62.4%, while for PCB77 and PCB81 congeners it was approx. 68.5% and 68.1%.

In the case of biodegradation of petroleum hydrocarbons (TPH) in sterile Soil B, strain *Mycolicibacterium frederiksbergense* IN53 showed the best degradability (37.2%), better than mixed culture M1 (33.6%). There was probably a similar situation to this in the case of soil A, and the effect of competition between strains for the same source of carbon and energy could occur. Then the *Rhodococcus* sp. IN306 turned out to be the weakest degrader, most likely due to specialization of its enzymatic apparatus to metabolize biphenyl and its derivatives.

Moreover, the obtained results of chromatographic analyses enabled determination of biodegradability of individual hydrocarbon groups (Table S2). The rate of individual hydrocarbon groups removal was arranged in a decreasing order nC_10_–nC_22_ > nC_23_–nC_28_ > nC_29_–nC_40_. This removal scheme is probably related to the chemical structure of alkanes. Alkanes with chain length C_10_–C_22_ are substances most willingly used by bacteria in metabolic processes [39,46,66,67]. For the most effective bacterial *Mycolicibacterium frederiksbergense* IN53, the content of aliphatic hydrocarbons with carbon chain length nC_10_–nC_22_ was reduced by 46.9%, and in the case of hydrocarbons with longer chains (nC_23_–nC_40_), the degree of their reduction was around 39.5%, which proves a high degree of their biodegradation [39,51].

The tests carried out on sterile Soil C (contaminated with PCBs and TPH) confirmed the previously obtained results and showed that the largest biodegradable potential against polychlorinated biphenyls was characterized by *Rhodococcus* sp. IN306, whereas against petroleum substances the strain *Mycolicibacterium frederiksbergense*. IN53 showed the largest potential. However, when it comes to the joint removal of both types of impurities, the mixed culture M1 proved to be the most effective. Probably in the case of soil polluted with a few (in this case two) xenobiotic types, the application of a bacterial mixed culture developed based on various (in this case three) bacterial strains allows to combine the degradation effect of all strains comprised by the mixed culture parallel to the reduction of competition between strains for the source of food (carbon contained in PCBs and hydrocarbon molecules).

In the case of non-sterile soil, there is always a justified risk that not every allochthonous strain will adapt equally well to the new environment, so there is a risk that the obtained biodegradation effect will be weaker [19,39,51]. In bioremediation techniques for historically PCBs polluted soil, it is preferred to apply mixed bacterial cultures [18,19,38,68]. The studies carried out now on acceleration of petroleum hydrocarbons (TPH) biodegradation through biotechnological processes, with the use of active bacterial cultures, prove that these are the most rational methods for bioremediation of polluted soil and due to relatively low costs and high effectiveness they are practically used on a technical scale [39,40,69].

Therefore, tests on the biodegradation process of PCBs and TPH were carried out in real Soil D (non-sterile), contaminated with petroleum derivatives and aged transformer oil, in ex-situ semi-technical prism method. As a result of the inoculation process with mixed culture M1 of non-sterile Soil D after a period of 6 months, the PCBs content was reduced by 84.5% and TPH by 70.8%.

The calculated first-order biodegradation constants (k) for inoculation with mixed culture M1 for non-sterile Soil D (Table S3) are on a level proving a satisfactory degree of individual PCB congeners biodegradation, decreasing with the increase in the number of chlorine atoms in a molecule [64]. Instead, for petroleum pollutants, they show higher biodegradability of aliphatic hydrocarbons with a carbon chain length nC_10_–nC_22_ as compared with heavier homologous (nC_23_–C_40_) and are close to results of studies presented in the world literature [39,46,67].

To assess the effectiveness of bioremediation, it is preferable to conduct toxicological monitoring using biotests belonging to different trophic groups: Microtox STP [70,71,72,73] Ostracodtoxkit [74,75], Phytotoxkit [76,77,78], MARA environmental risk assessment test [77,79,80,81,82] and the Muta-Chromoplate test based on the Ames test [39,83,84]. In this study, unlike other authors, extensive toxicological monitoring was conducted using a set of five biotests, whose bioindicators represented all trophic groups: reducers, decomposers, and producers. It allowed not only the observation of changes in toxicity in Soil D during the inoculation process with the mixed culture M1, but also the determination of the diverse toxicity of the tested organisms to contaminants contained in the tested soil. The calculated toxicity (TU) for individual tests was in decreasing order: Phytotoxkit < Microtox SPT < Ostracodtoxkit < MARA, which shows that the most sensitive bioindicators for impurities contained in the tested Soil D (TPH and PCBs) are *Heterocypris incongruens* and MARA microorganisms. As a result of the conducted bioremediation process, Soil D’s toxicity (TU) was reduced from the level of 19.6–10.5 to 5.2–3.1, while the mutagenicity coefficient from 9.85 to 0.68 proves that contaminated soil can be classified as low-toxic (Figure 8 and Figure 9).

The presented results clearly confirmed the rightness of the adopted bioremediation concept of soils contaminated with polychlorinated biphenyls (PCBs) in the presence of total petroleum hydrocarbon (TPH).

## 4. Materials and Methods

### 4.1. Soil and Microorganisms

To determine biodegradation effects of each strain, acting independently or within a mixed culture in the absence of native microbiota, three types of sterile soils were used in this studies. Namely, Soil A, commercially available PCB-polluted soil (Certified Reference Material—PCB Congeners in Soil, Product ID: SQC068-50g, Sigma-Aldrich, Laramie, WY, USA–Certificate of analysis Soil A contains in Supplementary Materials); Soil B, petroleum hydrocarbon polluted soil collected from the G-44 waste pit situated in southern-eastern Poland (N 54°14′55″, E 46°68′51″); and Soil C, soil polluted with both PCBs and TPH. Soil B and Soil C were sterilized in an autoclave (SMS ASVE) at 15 psi and 121 °C for 20 min. To determine biodegradation effects of mixed culture M1 in the presence of native microbiota, the ex-situ prism method was applied. A non-sterile Soil D polluted with both PCB and TPH (soil originating from the G-44 waste pit polluted with spent transformer oil containing PCB) was used.

The following bacterial strains—*Mycolicibacterium frederiksbergense* IN53 (formerly *Mycobacterium frederiksbergense* IN53), *Rhodococcus erythropol* is IN129, and *Rhodococcus* sp. IN306 were used in this study. They came from the hydrocarbon-degrading microbial collection of the Department of Microbiology (at the Oil and Gas Institute–National Research Institute, Krakow, Poland) and were isolated from temperate (IN129 and IN53) and desert (IN306) hydrocarbon-exposed soils. In addition, IN53 was previously tested alone or as a member of a hydrocarbon-degrading mixed culture [51]. Diagnostic features of the strain were determined based on microscopic observations, morphology, growth on selective agar media, and biochemical profile (API-Coryne test, bioMerieux). The strains were phylogenetically identified by sequencing of gene encoding for 16S rRNA as described previously [39,85].

Hydrocarbon-degrading capabilities towards biphenyl and other compounds (n-alkanes, pristane, and aromatic hydrocarbons) were examined using the method of Wrenn and Venosa [86] and by microscopic observations of strain growth on a Kievskaya mineral medium (1 g L^−1^ K_2_HPO_4_, 1 g L^−1^ KH_2_PO_4_, 1 g L^−1^ NH_4_NO_3_, 0.02 g L^−1^ CaCl_2_, 0.05 g L^−1^ FeCl_3_, 0.2 g L^−1^ MgSO_4_, 1 g L^−1^ NaCl, final pH 7.0 ± 0.2) supplemented with biphenyl (1% (*w*/*v*)). After 30-day incubation at room temperature, the strain growth was verified by microscopic observations.

Pure cultures of the individual strains were grown in BD Difco™ nutrient broth (Difco, USA) supplemented with sodium acetate (0.2% (*w*/*v*), POCH, Poland), incubated at room temperature (25 °C) with shaking at 150 rpm for 3–7 days. They reached a density of 3.6 ± 2.4 × 10^8^ (IN53), 8.4 ± 3.15 × 10^8^ (IN129) and 4.1 ± 4.8 × 10^8^ (IN306) colony forming units/mL (CFU·ml^−1^), respectively, as judged by direct counts on BD Difco™ nutrient agar with sodium acetate (0.2% *w*/*v*). Microbial mixed culture M1 was constructed by mixing equal volumes of each strain (1:1:1) and, therefore, the obtained density was presumable 1 × 10^8^–1 × 10^9^ CFU·mL^−1^.

### 4.2. Experimental Setup

#### 4.2.1. Respirometric Tests

The respirometric tests based on the measurement of consumed oxygen (O_2_) consumption and/or of released carbon dioxide (CO_2_) allow to determine possibilities of aerobic biodegradation of the analyzed soil pollutants. Based on the O_2_ consumption and/or CO_2_ production rate, it is possible to estimate the rate of aerobic biodegradation of organic pollutant, i.e., the rate of substrate loss over time. Moreover, the results of respirometric tests illustrate the degree of metabolic activity of the polluted environment [87].

Studies on biodegradation of PCBs (Soil A), TPH (Soil B), and both PCBs and TPH (Soil C) in sterile soils were carried out in an Oxi-Top^®^ system (WTW, Bartoszyce, Poland). Twenty grams of each sterile soil were placed in glass sample cells; the system humidity approached the level of 25%. Inoculation was performed using 2 mL of previously isolated bacterial strains IN53, IN129, and IN309 with a density of 1 × 10^8^–1 × 10^9^ CFU·mL^−1^ and mixed culture M1. At the same time, control samples were prepared. In respirometric tests, control samples were sterile, and uncontaminated “pure” soil samples were inoculated with IN53, IN129, IN306 bacterial stains and mixed culture M1. Control samples were prepared analogously to test samples. After the inoculation, the sample cells were tightly closed with measuring heads, placed in an incubator and thermostated at 20 °C for 30 days. Measuring heads of the Oxi-Top Control system were reading the value of the pressure in the system every 2 h. The acquired data by means of an IR interface was transferred to the Oxi-Top OC 110 controller, where it could be graphically and statistically processed by means of the Achat OC software (WTW, Wroclaw, Poland) [88]. The measured pressure was converted into the amount of used oxygen (MO_2_) according to Equation (1) [89]. After the test completion (30 days), analytes were extracted from the studied soils for further analysis by means of gas chromatography. All experiments were performed three times with five repetitions in each treatment.

(1) $$m_{O2}=\frac{M(O_{2})}{RT_{m}}\cdot (V_{g}+\alpha \frac{T_{m}}{T_{0}})\cdot \Delta p$$

where: *M*(*O*_2_)—molar mass of oxygen [kg/mol], *V_g_*—free gas volume [m^3^], *R*—gas constant [J mol^−1^ K^−1^], *T_m_*—measured value of temperature [K], *T*_0_—reference temperature (273.15 K), *α*—absorption coefficient (0.03103), and Δ*p*—pressure drop in the test [Pa].

#### 4.2.2. PCBs and TPH Biodegradation in Non-sterile Soil-the Ex-Situ Prism Method

Studies on PCBs and TPH biodegradation in non-sterile soil (Soil D) were carried out by means of the ex-situ prism method. The soil at an amount of 50 kg was placed on a specially designed test stand, ensuring constant temperature (20–25 °C) and humidity (20–25%) during the experiment (Figure S2). A proper choice of biogenic substances and determination of their concentrations in the soil prior to starting the bioremediation is extremely important and allows to enhance the environment with nutrients and to optimize parameters of the process course, where for proper activity of microorganisms, the C:N:P ratio should be approx. 100:10:1 [39,46,67]. The soil pH was corrected, supplementing fertilizer lime at an amount of 1.0–1.5 g/kg of soil till obtaining the optimum pH 7.5–7.6. Prior to the soil inoculation, nitrogen and phosphorus were supplemented in the form of Azofoska mineral fertilizer as follows: 13.6% of total N, 5.5% of nitrate nitrogen, 8.1% of ammonia nitrogen, 6.4% of soluble P_2_O_5_, 19.1% of K_2_O in the form of K_2_SO_4_, 4.4% of MgO in the form of soluble MgSO_4_, as well as microelements (0.17% Fe, 0.27% Mn, 0.18% Cu, 0.045% Zn, 0.09% Mo) to obtain optimum proportions of nutrients C:N:P = 100:10:1. Nutrients (nitrogen and phosphorous, present in the soil as ions: NH_4_-N (Merck, Spectroquant^®^ cat. no. 114752, Darmstadt, Germany), NO_3_-N (Merck, Spectroquant^®^ cat. No. 109713), and PO_4_-P (Merck, Spectroquant^®^ cat. No. 114543) were determined by Perkin Elmer Lambda 40 spectrophotometer. Before the inoculation, the total number of heterotrophic aerobic bacteria in non-sterile soil D was 5.9 ± 5.1·10^5^ CFU·g^−1^ dry mass. The soil prepared in this way was inoculated with mixed culture M1 with a density of 10^9^ CFU mL^−1^ (2 L mixed culture M1/50 kg soil D). After 6 months of the experiment, the number of bacteria in D6 soil was 4.1 ± 4.6 × 10^7^ CFU·g^−1^. The PCBs and TPH biodegradation was monitored by means of chromatographic analyses (every 30 days during 6 months) and ecotoxicological tests. Control was non-sterile Soil D non-inoculated with mixed culture M1.

### 4.3. Chromatographic Analysis

#### 4.3.1. PCB Extraction and Quantification

Ten grams of dried soil were placed in an Erlenmeyer flask and extracted using a mixture (80 mL) of n-hexane and dichloromethane (POCH S.A., Gliwice, Poland) at 1:1 ratio [90]. The PCBs were extracted on a Conbest DOS-20L shaker for 3 h, at a constant temperature of 30 °C and shaking rate of 150 rpm [91]. After 3 h, 1 g of powdered copper (Merck, Darmstadt, Germany) was added to the extract and the mixture was extracted for another hour [19]. Copper and interfering substances were removed via filtration using a set of Backerbond columns (silica gel No 7086-03 and PCB-A No 7511-04). The solvent was evaporated in a vacuum rotary evaporator and the extract was dissolved in 1 mL of isooctane (Merck, Germany) and analyzed by the GC method. An internal PCB-209 standard (2,2′,3,3′,4,4′,5,5′,6,6′-decachlorobiphenyl) (Sigma-Aldrich, USA) was used to determine the extraction yield with recovery of approx. 93%.

PCB congeners were analyzed by means of gas chromatography (GC), using a Perkin Elmer Clarus 500 chromatograph with an electron capture detector (ECD). The conditions of GC chromatograph operation were as follows: a capillary column of fused silica (Rtx-XLB 30 m × 0.32 mm ID) (Restek, Bellefonte, PA, USA) with a low-polarity proprietary phase (0.50 µm thick), with the application of the following temperature parameters: injector temperature 280 °C; ECD detector temperature 320 °C; oven temperature programme: 120 °C (2 min isothermal run), 120–280 °C (temperature increase 15 °C min^−1^), 280 °C (5 min isothermal run), 280–320 °C (temperature increase 28 °C min^−1^), and 320 °C (10 min isothermal run). The carrier gas was helium (He) at a constant flow of 3.5 mL·min^−1^. The identification and quantitative analyses of 18 PCB congeners (PCB28 (2,4,4′-), PCB52 (2,2′,5,5′-), PCB77 (3,3′,4,4′-), PCB81 (3,4,4′,5-), PCB101 (2,2′,4,5,5′-), PCB105 (2,3,3′,4,4′-), PCB114 (2,3,4,4′,5-), PCB118 (2,3′,4,4′,5-), PCB123 (2,3′,4,4′,5′-), PCB126 (3,3′,4,4′,5-), PCB138 (2,2′,3,4,4′,5′-), PCB 153 (2,2′,4,4′,5,5′-), PCB156 (2,3,3′,4,4′,5-), PCB157 (2,3,3′,4,4′,5-), PCB167 (2,3′,4,4′,5,5′-), PCB169 (3,3′,4,4′,5,5′-), PCB180 (2,2′,3,4,4′,5,5′-), and PCB189 (2,3,3′,4,4′,5,5′-) were carried out using a certified reference material namely: PCB congeners in the SQC068-50g soil (Sigma-Aldrich, USA). A certified PCB-209 standard (Sigma-Aldrich, Buchs, Switzerland) was used as a biomarker.

#### 4.3.2. TPH Extraction and Quantification

Ten grams of dried soil were placed in an Erlenmeyer flask and extracted using dichloromethane (POCH S.A., Poland) in three series (20 mL of solvent, 15 min). The extraction of petroleum hydrocarbons was carried out by means of the sonification method in an ultrasonic bath Sonoswiss SW 6H, at an ultrasonic frequency of 30 kHz [92]. Polar substances were removed by filtration through Backerbond columns with Florisil No 7213-03 packing. The solvent was evaporated in a vacuum rotary evaporator and the extract was dissolved in 1 mL of dichloromethane and analysed by the GC method. A substitute standard o-tertphenyl was used to determine the extraction yield with recovery of approx. 95.9% [39].

TPH were analysed by means of gas chromatography (GC) using a Perkin Elmer Clarus 500 GC chromatograph with a flame ionisation detector (FID). The conditions of chromatograph operation were as follows: a capillary column of fused silica (RTX-1: 30 m × 0.53 mm) (Restek, USA), applying the following temperature parameters: injector temperature = 290 °C, FID detector temperature = 320 °C, oven temperature programme: 30 °C (2 min. isothermal run), 30–105 °C (temperature increase 10 °C min^−1^), 105–285 °C (temperature increase 5 °C min^−1^), and 285 °C (5 min isothermal run). The carrier gas was He at a constant flow of 20 mL·min^−1^. For the quantitative determination of total petroleum pollutants content (TPH), a set of Tusnovic Instruments calibration standards was used (certified standard: BAM K010). In turn, certified sets of Supelco and Restek standards (standard mixture No D2807 of paraffin hydrocarbons: nC_6_–nC_44_ and certified standard mixture No A029668: Fuel Oil Degradation Mix n-C_17_, pristane, n-C_18_, phytane) were used to quantitatively determine individual n-alkanes being components of petroleum pollutants. A certified standard C_30_ 17α(H), 21β(H)-hopane No 08189 (Sigma-Aldrich, Switzerland) was used as a biomarker.

### 4.4. Mathematical Model of PCB and TPH Biodegradation

A simplified mathematical model of polychlorinated biphenyls and petroleum hydrocarbons biodegradation during the performance of tests by the ex-situ method, was developed. PCB-209 biomarker (Sigma-Aldrich, USA) was applied to normalize concentrations of the following analytes: PCBs, TriCB, TetraCBs, PentaCBs, HexaCBs, and HeptaCBs. To normalize concentrations of: TPH, ΣnC_10_–nC_22_, and ΣnC_23_–nC_40_, a biomarker C_30_17α(H),21β(H)-hopane (Supelco, Bellefonte, PA, USA) was used. The biomarker application of biomarkers in biodegradation studies allows to eliminate analytical errors in chromatographic determinations of individual analyte groups. Normalized values were used to develop a mathematical first-order model describing biodegradation course, according to Equation (2) [39,64].

C/C_x_ = (C/C_x_)_0_ exp (−kt) (2)

where: C—analyte concentration, C_x_—biomarker (PCB-209 or Hopane) concentration, k—first-order rate constant [d^−1^], (C/C_x_)_0_—normalized analyte concentration at the starting point, and t—process duration [d].

The analysis of non-linear regression using Equation (2) enabled determination of the first-order biodegradation constant (k) and the correlation coefficient (r^2^) defining the measuring points fit to theoretical curves of the biodegradation process: PCBs, TriCB, TetraCBs, PentaCBs, HexaCBs, HeptaCBs, TPH, ΣnC_11_–nC_22_, and ΣnC_23_–nC_40_.

### 4.5. Ecotoxicological Analyses

Petroleum hydrocarbon biodegradation leads to the formation of various metabolites often with poorly recognized biological activity. Because of that, it is recommended to carry out toxicological tests, which enable monitoring the changes of soil toxicity. In this study, assessment of toxicity changes during PCB and TPH biodegradation in a non-sterile soil (soil D) inoculated with mixed culture M1 (the ex-situ prism method) was performed. A set of new generation tests, which bioindicators belong to various trophic levels, was used. The specification of toxicological tests used in this studies is presented in Table 2, while the description of their performance procedures is presented in Supplementary Materials.

### 4.6. Data Analysis and Statistical Information

Statistical analysis was performed with Statistica 13.3 (StatSoft, Krakow, Poland). Standard deviation (SD), relative standard deviation (RSD) (%) and Pearson correlation coefficient were calculated. Statistically significant differences were evaluated with a one-way ANOVA followed by a post-hoc pairwise Tukey test (when the ANOVA produced significant results). Significance was set at *p* < 0.05.

## 5. Conclusions

The study was aimed at the assessment of bacterial strains application possibilities: *Mycolicibacterium frederiksbergense* IN53, *Rhodococcus erythropolis* IN129, *Rhodococcus* sp. IN306, and mixed culture M1 developed based on these strains. The results of both respirometric tests and chromatographic analyses revealed that chosen bacterial strains with potential PCB decomposition capacity are actually capable of removing this type of pollution from the soil, as well as they are capable of decomposing petroleum hydrocarbons (in particular aliphatic ones). *Rhodococcus* sp. IN306 appeared as the most effective PCB-degrader among the studied strains. Moreover, IN306 is very closely related to *Rhodococcus jostii* RHA-1 (bacterial strains with great PCB-transforming potential). It was shown that the degree of PCB congeners biodegradation was decreasing with increasing number of chlorine atoms and depended on their spatial structure. Petroleum pollutants, in particular n-alkanes, were degraded by *Mycolicibacterium frederiksbergense* IN53 in the most efficient way. Its effect on low-, medium- (nC_10_–nC_22_) and long-chain compounds (nC_23_–nC_40_) was the greatest among all tested bioaugmentation variants. In turn, mixed culture M1 developed on the basis of tested bacterial strains, featured satisfactory biodegradation capacity both for individual PCB congeners and TPH. The action of mixed culture M1 was verified in a semi-technical scale experiment under ex-situ conditions on a soil polluted with aged petroleum hydrocarbons (TPH) as well as spent transformer oil (PCBs). The effectiveness of this treatment was confirmed by the results of both chromatographic and toxicological tests.

## Figures and Tables

**Table 1:** Genes involve in biphenyl and hydrocarbon degradation, found in genomes of the closest relatives of IN53, IN129 and IN306.

Strain	The Closest Relative Based on 16S rRNA Accession Number, (% of Identity) *	The Closest Relative for Which the Genome Sequence Is Available in NCBI GenBank, Accession Number, (% of Identity)	Gene Encoding for the Enzymes Degrading Hydrocarbons
*Mycolicibacterium frederiksbergense* IN53 JN572675	*Mycolicibacterium frederiksbergense* DSM 44346 (typical strain) NR_025393.1 99.58%	*Mycobacterium* sp. YC-RL4, CP015596, (99.24%)	Alkane 1-monooxygenase (2 copies), pentachlorophenol monooxygenase, 2,3-dihydroxybiphenyl 1,2-dioxygenase (2 copies), ring-hydroxylating dioxygenase alpha subunit
*Rhodococcus erythropolis* IN129 KT923311	*Rhodococcus erythropolis* IN104 KT923338 100%	*Rhodococcus erythropolis* X5, CP044284, (99.93%)	Alkane 1-monooxygenase (5copies), pentachlorophenol monooxygenase, phenol/toluene hydroxylase, biphenyl 2,3-dioxygenase, 2,3-dihydroxybiphenyl 1,2-dioxygenase (3 copies), ring-hydroxylating dioxygenase alpha subunit
*Rhodococcus* sp. IN306 KX058399	*Rhodococcus* sp. S2-17 KY765341 99.30%	*Rhodococcus jostii* RHA1, CP000431, (98.75%)	Alkane 1-monooxygenase, pentachlorophenol monooxygenase, phenol/toluene hydroxylase, biphenyl 2,3-dioxygenase, 2,3-dihydrodihydroxybiphenyl 2,3-dehydrogenase, 2,3-dihydroxybiphenyl 1,2-dioxygenase (5 copies),

* the presented data concerns the data gathered in the NCBI GenBank database (November 2019).

**Table 2:** Tests used in monitoring of toxicity alternations in soil under biodegradation.

Trophic Level	Life Form	Test Type		Test Reaction	Reference
Consumers	*Heterocypris incongruens*	Ostracodtoxkit(F)^TM^ (MicroBioTest Inc., Gent, Belgium)		growth inhibition, mortality	[74,75]
Producers	*Sorghum saccharatum* *Sinapis alba* *Lepidium sativum*	Phytotoxkit^TM^ test (MicroBioTests Inc., Belgium),		growth inhibition, germinated seeds	[76,77,78]
Decomposers	*Vibrio fischeri*	Microtox^®^ Solid Phase Test (SDI, New Castle, DE, USA)		luminescence inhibitionTU = 100/EC_50_	[70,71,72,73]
Decomposers	*Set of 11 bacteria strains*	MARA environmental risk test		growth inhibition	[77,79,80,81,82]
		oil contaminants were extracted (SPE) and vaporised, deposit was dissolved in dimethylsulfooxygen (DMSO)			
	*Microbacterium* sp., *Brevundimonas diminuta, Citrobacter freudii, Comamonas testosterone, Enterococcus casseliflavus, Delftia acidovorans, Kurthia gibsoni, Staphylococcus warnerii, Pseudomonas aurantiaca, Serratia rubidaea, Pichia anomala)*				
Decomposers	*Salmonella typhimurium* (*TA*-*98*, *TA*-*100*)		AMES (Muta-ChromoPlate^TM^ Kit) (EBPI, Mississauga, ON, Canada).	revertants number	[39,83,84]
			oil contaminants were extracted (SPE) and vaporised, deposit was dissolved in DMSO		

## Supplementary Materials

The following are available online, Table S1: Comparison of PCB content in sterile soil samples A and C after inoculation with *Mycolicibacterium frederiksbergense* IN53, *Rhodocococcus erythropolis* IN129, *Rhodococcus* sp. IN306 and mixed culture M1 over 30 days. Measurement repetition number *n* = 8–10, <0.05., Table S2: Comparison of TPH, unidentified hydrocarons, alkanes (ΣnC10–nC22, ΣnC23–nC40 content in sterile soil samples B and C after by *Mycolicibacterium frederiksbergense* IN53, *Rhodococcus erythropolis* IN129, *Rhodococcus* sp. IN306 and mixed culture M1 after the 30 days biodegradation process. Measurement repetition number *n* = 8–10, <0.05, Table S3: Coefficients of first-order mathematical model describing PCB and TPH pollutants group biodegradation in non-sterile soil D (ex-situ prism method). Measurement repetition number *n* = 7–10, <0.05, Figure S1: Percentage share of identified pollutants in the sample (Soil D) comprising: (a) PCBs, (b) TPH, Figure S2: Scheme of the test stand for conducting the process of biodegradation of pollutants (PCBs and TPH) in soil D under semi-technical conditions (ex situ prism method), Certificate of Analysis Soil A, Ecotoxicological analyses–description of the methodology.

## References

[1] Field J.A., Sierra-Alvarez R. (2008). Microbial transformation and degradation of polychlorinated biphenyls. Environ. Pollut..

[2] Vasilyeva G.K., Strijakova E.R. (2007). Bioremediation of soils and sediments contaminated by polychlorinated biphenyls. Microbiology.

[3] Pieper D.H., Seeger M. (2008). Bacterial metabolism of polychlorinated biphenyls. J. Mol. Microbiol. Biotechnol..

[4] Furukawa K., Fujihara H. (2008). Microbial degradation of polychlorinated biphenyls: Biochemical and molecular features. J. Biosci. Bioeng..

[5] Correa P.A., Lin L., Just C.L., Hu D., Hornbuckle K.C., Schnoor J.L., Van Aken B. (2010). The effects of individual PCB congeners on the soil bacterial community structure and the abundance of biphenyl dioxygenase genes. Environ. Int..

[6] Dudášová H., Lukáčová L., Murínová S., Puškárová A., Pangallo D., Dercová K. (2014). Bacterial strains isolated from PCB-contaminated sediments and their use for bioaugmentation strategy in microcosms. J. Basic Microbiol..

[7] Shaikh N.S., Parkin S., Luthe G., Lehmler H.-J. (2008). The three-dimensional structure of 3,3′,4,4′-tetrachlorobiphenyl, a dioxin-like polychlorinated biphenyl (PCB). Chemosphere.

[8] Erickson M.D., Kaley R.G. (2011). Applications of polychlorinated biphenyls. Environ. Sci Pollut. Res..

[9] Borja J., Taleon D.M., Auresenia J., Gallardo S. (2005). Polychlorinated biphenyls and their biodegradation. Process Biochem..

[10] Sharma J.K., Gautam R.K., Nanekar S.V., Weber R., Singh B.K., Singh S.K., Juwarkar A.A. (2018). Advances and perspective in bioremediation of polychlorinated biphenyl-contaminated soils. Environ. Sci Pollut. Res..

[11] Blanco-Moreno R., Sáez L.P., Luque-Almagro V.M., Roldán M.D., Moreno-Vivián C. (2017). Isolation of bacterial strains able to degrade biphenyl, diphenyl ether and the heat transfer fluid used in thermo-solar plants. New Biotechnol..

[12] Liang Y., Martinez A., Hornbuckle K.C., Mattes T.E. (2014). Potential for polychlorinated biphenyl biodegradation in sediments from Indiana Harbor and Ship Canal. Int. Biodeterior. Biodegrad..

[13] Mukerjee-Dhar G. (2005). *Bph* genes of the thermophilic PCB degrader, *Bacillus* sp. JF8: Characterization of the divergent ring-hydroxylating dioxygenase and hydrolase genes upstream of the Mn-dependent BphC. Microbiology.

[14] Suyamud B., Inthorn D., Panyapinyopol B., Thiravetyan P. (2018). Biodegradation of bisphenol A by a newly isolated *Bacillus megaterium* strain ISO-2 from a polycarbonate industrial wastewater. Water Air Soil Pollut..

[15] Ponce B.L., Latorre V.K., González M., Seeger M. (2011). Antioxidant compounds improved PCB-degradation by *Burkholderia xenovorans* strain LB400. Enzym. Microb. Technol..

[16] Sierra I., Valera J.L., Marina M.L., Laborda F. (2003). Study of the biodegradation process of polychlorinated biphenyls in liquid medium and soil by a new isolated aerobic bacterium (*Janibacter* sp.). Chemosphere.

[17] Moody J., Doerge D., Freeman J., Cerniglia C. (2002). Degradation of biphenyl by *Mycobacterium* sp. strain PYR-1. Appl. Microbiol. Biotechnol..

[18] Murínová S., Dercová K. (2014). Potential Use of Newly Isolated Bacterial Strain *Ochrobactrum anthropi* in bioremediation of polychlorinated biphenyls. Water Air Soil Pollut..

[19] Horváthová H., Lászlová K., Dercová K. (2018). Bioremediation of PCB-contaminated shallow river sediments: The efficacy of biodegradation using individual bacterial strains and their consortia. Chemosphere.

[20] Zorádová S., Dudášová H., Lukáčová L., Dercová K., Čertík M. (2011). The effect of polychlorinated biphenyls (PCBs) on the membrane lipids of Pseudomonas stutzeri. Int. Biodeterior. Biodegrad..

[21] Tao B., Zhou Y., He X., Li D. (2014). *Pseudomonas chengduensis* sp. nov., isolated from landfill leachate. Int. J. Syst. Evol. Microbiol..

[22] Ridl J., Suman J., Fraraccio S., Hradilova M., Strejcek M., Cajthaml T., Zubrova A., Macek T., Strnad H., Uhlik O. (2018). Complete genome sequence of *Pseudomonas alcaliphila* JAB1 (=DSM 26533), a versatile degrader of organic pollutants. Stand Genom. Sci..

[23] Chakraborty J., Das S. (2016). Characterization of the metabolic pathway and catabolic gene expression in biphenyl degrading marine bacterium *Pseudomonas aeruginosa* JP-11. Chemosphere.

[24] Suenaga H., Fujihara H., Kimura N., Hirose J., Watanabe T., Futagami T., Goto M., Shimodaira J., Furukawa K. (2017). Insights into the genomic plasticity of *Pseudomonas putida* KF715, a strain with unique biphenyl-utilizing activity and genome instability properties: Genomic plasticity of *Pseudomonas putida* KF715. Environ. Microbiol. Rep..

[25] Weiland-Bräuer N., Fischer M.A., Schramm K.-W., Schmitz R.A. (2017). Polychlorinated biphenyl (PCB)-degrading potential of microbes present in a cryoconite of Jamtalferner Glacier. Front. Microbiol..

[26] Chung S.-Y., Maeda M., Song E., Horikoshij K., Kudo T. (1994). A Gram-positive polychlorinated biphenyl-degrading bacterium, *Rhodococcus erythropolis* strain TA421, isolated from a termite ecosystem. Biosci. Biotechnol. Biochem..

[27] Taguchi K., Motoyama M., Kudo T. (2004). Multiplicity of 2,3-dihydroxybiphenyl dioxygenase genes in the gram-positive polychlorinated biphenyl degrading bacterium *Rhodococcus rhodochrous* K37. Biosci. Biotechnol. Biochem..

[28] Murínová S., Dercová K., Dudášová H. (2014). Degradation of polychlorinated biphenyls (PCBs) by four bacterial isolates obtained from the PCB-contaminated soil and PCB-contaminated sediment. Int. Biodeterior. Biodegrad..

[29] Pham T.T.M., Pino Rodriguez N.J., Hijri M., Sylvestre M. (2015). Optimizing polychlorinated biphenyl degradation by flavonoid-induced cells of the *Rhizobacterium Rhodococcus erythropolis* U23A. PLoS ONE.

[30] Shumkova E.S., Olsson B.E., Kudryavtseva A.V., Plotnikova E.G. (2015). Draft genome sequence of *Rhodococcus ruber* strain P25, an active polychlorinated biphenyl degrader. Genome Announc..

[31] Atago Y., Shimodaira J., Araki N., Bin Othman N., Zakaria Z., Fukuda M., Futami J., Hara H. (2016). Identification of novel extracellular protein for PCB/biphenyl metabolism in *Rhodococcus jostii* RHA1. Biosci. Biotechnol. Biochem..

[32] Xu Y., Yu M., Shen A. (2016). Complete genome sequence of the polychlorinated biphenyl degrader *Rhodococcus* sp. WB1. Genome Announc..

[33] Wang H., Hu J., Xu K., Tang X., Xu X., Shen C. (2018). Biodegradation and chemotaxis of polychlorinated biphenyls, biphenyls, and their metabolites by *Rhodococcus* sp.. Biodegradation.

[34] Sato T., Todoroki T., Shimoda K., Terada A., Hosomi M. (2010). Behavior of PCDDs/PCDFs in remediation of PCBs-contaminated sediments by thermal desorption. Chemosphere.

[35] Wang X., Teng Y., Luo Y., Dick R.P. (2016). Biodegradation of 3,3′,4,4′-tetrachlorobiphenyl by *Sinorhizobium meliloti* NM. Bioresour. Technol..

[36] Jing R., Fusi S., Kjellerup B.V. (2018). Remediation of polychlorinated biphenyls (PCBs) in contaminated soils and sediment: State of knowledge and perspectives. Front. Environ. Sci..

[37] Pathiraja G., Egodawatta P., Goonetilleke A., Teo V.S.J. (2019). Effective degradation of polychlorinated biphenyls by a facultative anaerobic bacterial consortium using alternating anaerobic aerobic treatments. Sci. Total Environ..

[38] Cervantes-González E., Guevara-García M.A., García-Mena J., Ovando-Medina V.M. (2019). Microbial diversity assessment of polychlorinated biphenyl–contaminated soils and the biostimulation and bioaugmentation processes. Environ. Monit. Assess.

[39] Steliga T., Jakubowicz P., Kapusta P. (2012). Changes in toxicity during in situ bioremediation of weathered drill wastes contaminated with petroleum hydrocarbons. Bioresour. Technol..

[40] Silva-Castro G.A., Rodriguez-Calvo A., Laguna J., González-López J., Calvo C. (2016). Autochthonous microbial responses and hydrocarbons degradation in polluted soil during biostimulating treatments under different soil moisture. Assay in pilot plant. Int. Biodeterior. Biodegrad..

[41] Roy A., Dutta A., Pal S., Gupta A., Sarkar J., Chatterjee A., Saha A., Sarkar P., Sar P., Kazy S.K. (2018). Biostimulation and bioaugmentation of native microbial community accelerated bioremediation of oil refinery sludge. Bioresour. Technol..

[42] Sarkar P., Roy A., Pal S., Mohapatra B., Kazy S.K., Maiti M.K., Sar P. (2017). Enrichment and characterization of hydrocarbon-degrading bacteria from petroleum refinery waste as potent bioaugmentation agent for in situ bioremediation. Bioresour. Technol..

[43] Tyagi M., da Fonseca M.M.R., de Carvalho C.C.C.R. (2011). Bioaugmentation and biostimulation strategies to improve the effectiveness of bioremediation processes. Biodegradation.

[44] Havel J., Reineke W. (1992). Degradation of Aroclor 1221 and survival of strains in soil microcosms. Appl Microbiol. Biotechnol..

[45] Egorova D.O., Demakov V.A., Plotnikova E.G. (2013). Bioaugmentation of a polychlorobiphenyl contaminated soil with two aerobic bacterial strains. J. Hazard. Mater..

[46] Wu M., Li W., Dick W.A., Ye X., Chen K., Kost D., Chen L. (2017). Bioremediation of hydrocarbon degradation in a petroleum-contaminated soil and microbial population and activity determination. Chemosphere.

[47] Lászlová K., Dudášová H., Olejníková P., Horváthová G., Velická Z., Horváthová H., Dercová K. (2018). The application of biosurfactants in bioremediation of the aged sediment contaminated with polychlorinated biphenyls. Water Air Soil Pollut..

[48] Passatore L., Rossetti S., Juwarkar A.A., Massacci A. (2014). Phytoremediation and bioremediation of polychlorinated biphenyls (PCBs): State of knowledge and research perspectives. J. Hazard. Mater..

[49] Steliga T., Kapusta P., Jakubowicz P. (2009). Effectiveness of bioremediation processes of hydrocarbon pollutants in weathered drill wastes. Water Air Soil Pollut..

[50] Steliga T., Jakubowicz P., Kapusta P., Kluk D. (2018). Badania biodegradacji odpadów wiertniczych zanieczyszczonych substancjami ropopochodnymi (ang. Study on biodegradation of drilling wastes contaminated with petroleum hydrocarbons). Przemysł Chem..

[51] Brzeszcz J., Steliga T., Kapusta P., Turkiewicz A., Kaszycki P. (2016). r-strategist versus K-strategist for the application in bioremediation of hydrocarbon-contaminated soils. Int. Biodeterior. Biodegrad..

[52] Pruesse E., Peplies J., Glöckner F.O. (2012). SINA: Accurate high-throughput multiple sequence alignment of ribosomal RNA genes. Bioinformatics.

[53] Ludwig W., Strunk O., Westram R., Richter L., Meier H., Yadhukumar, Buchner A., Lai T., Steppi S., Jobb G. (2004). ARB: A software environment for sequence data. Nucleic Acids Res..

[54] Elangovan S., Pandian S.B.S., Geetha S.J., Joshi S.J., Arora P.K. (2019). Polychlorinated biphenyls (PCBs): Environmental fate, challenges and bioremediation. Microbial Metabolism of Xenobiotic Compounds.

[55] Tu C., Teng Y., Luo Y., Li X., Sun X., Li Z., Liu W., Christie P. (2011). Potential for biodegradation of polychlorinated biphenyls (PCBs) by *Sinorhizobium meliloti*. J. Hazard. Mater..

[56] Horváthová H., Lászlová K., Dercová K. (2019). Bioremediation vs. nanoremediation: Degradation of polychlorinated biphenyls (PCBS) using integrated remediation approaches. Water Air Soil Pollut..

[57] Nabavi B., Nikaeen M., Amin M., Hatamzadeh M. (2013). Isolation and identification of aerobic polychlorinated biphenyls degrading bacteria. Int. J. Environ. Health Eng..

[58] McLeod M.P., Warren R.L., Hsiao W.W.L., Araki N., Myhre M., Fernandes C., Miyazawa D., Wong W., Lillquist A.L., Wang D. (2006). The complete genome of *Rhodococcus* sp. RHA1 provides insights into a catabolic powerhouse. Proc. Natl. Acad. Sci. USA.

[59] Rodrigues J.L.M., Kachel C.A., Aiello M.R., Quensen J.F., Maltseva O.V., Tsoi T.V., Tiedje J.M. (2006). Degradation of Aroclor 1242 dechlorination products in sediments by *Burkholderia xenovorans* LB400(*ohb*) and *Rhodococcus* sp. Strain RHA1(*fcb*). Appl. Environ. Microbiol..

[60] Ohmori T., Morita H., Tanaka M., Miyauchi K., Kasai D., Furukawa K., Miyashita K., Ogawa N., Masai E., Fukuda M. (2011). Development of a strain for efficient degradation of polychlorinated biphenyls by patchwork assembly of degradation pathways. J. Biosci. Bioeng..

[61] McKay D.B., Prucha M., Reineke W., Timmis K.N., Pieper D.H. (2003). Substrate specificity and expression of three 2,3-dihydroxybiphenyl 1,2-dioxygenases from *Rhodococcus globerulus* Strain P6. J. Bacteriol..

[62] Yang X., Sun Y., Qian S. (2004). Biodegradation of seven polychlorinated biphenyls by a newly isolated aerobic bacterium (*Rhodococcus* sp. R04). J. Ind. Microbiol. Biotechnol..

[63] Hu C., Zhang Y., Tang X., Luo W. (2014). PCB biodegradation and *bphA1* gene expression induced by salicylic acid and biphenyl with *Pseudomonas fluorescence* P2W and *Ralstonia eutropha* H850. Pol. J. Environ. Stud..

[64] Zhang H., Jiang X., Lu L., Xiao W. (2015). Biodegradation of polychlorinated biphenyls (PCBs) by the novel identified *cyanobacterium Anabaena* PD-1. PLoS ONE.

[65] Furukawa K. (1994). Molecular genetics and evolutionary relationship of PCB-degrading bacteria. Biodegradation.

[66] Abbasian F., Lockington R., Megharaj M., Naidu R. (2016). A review on the genetics of aliphatic and aromatic hydrocarbon degradation. Appl. Biochem. Biotechnol..

[67] Nwankwegu A.S., Orji M.U., Onwosi C.O. (2016). Studies on organic and in-organic biostimulants in bioremediation of diesel-contaminated arable soil. Chemosphere.

[68] Dercová K., Čičmanová J., Lovecká P., Demnerová K., Macková M., Hucko P., Kušnír P. (2008). Isolation and identification of PCB-degrading microorganisms from contaminated sediments. Int. Biodeterior. Biodegrad..

[69] Guarino C., Zuzolo D., Marziano M., Conte B., Baiamonte G., Morra L., Benotti D., Gresia D., Stacul E.R., Cicchella D. (2019). Investigation and assessment for an effective approach to the reclamation of polycyclic aromatic hydrocarbon (PAHs) contaminated site: SIN Bagnoli, Italy. Sci. Rep..

[70] Adams R.H., Kanga-Leyva K., Guzmán-Osorio F.J., Escalante- Espinosa E. (2011). Comparison of moisture management methods for the bioremediation of hydrocarbon contaminated soil. Afr. J. Biotechnol..

[71] Foucault Y., Durand M.-J., Tack K., Schreck E., Geret F., Leveque T., Pradere P., Goix S., Dumat C. (2013). Use of ecotoxicity test and ecoscores to improve the management of polluted soils: Case of a secondary lead smelter plant. J. Hazard. Mater..

[72] Lima T.M.S., Procópio L.C., Brandão F.D., Leão B.A., Tótola M.R., Borges A.C. (2011). Evaluation of bacterial surfactant toxicity towards petroleum degrading microorganisms. Bioresour. Technol..

[73] Oleszczuk P., Jośko I., Kuśmierz M., Futa B., Wielgosz E., Ligęza S., Pranagal J. (2014). Microbiological, biochemical and ecotoxicological evaluation of soils in the area of biochar production in relation to polycyclic aromatic hydrocarbon content. Geoderma.

[74] Niyommaneerat W., Nakajima F., Tobino T., Yamamoto K. (2017). Development of a chronic sediment toxicity test using the benthic ostracod *Heterocypris incongruens* and their application to toxicity assessments of urban road dust. Ecotoxicol. Environ. Saf..

[75] Steliga T. (2011). The use of biotests in estimation of bioremediation processes in weathered drilling wastes. Arch. Environ. Prot..

[76] Baran A., Tarnawski M. (2013). Phytotoxkit/Phytotestkit and Microtox^®^ as tools for toxicity assessment of sediments. Ecotoxicol. Environ. Saf..

[77] Fai P.B., Grant A. (2010). An assessment of the potential of the microbial assay for risk assessment (MARA) for ecotoxicological testing. Ecotoxicology.

[78] Mamindy-Pajany Y., Hamer B., Roméo M., Géret F., Galgani F., Durmiši E., Hurel C., Marmier N. (2011). The toxicity of composted sediments from Mediterranean ports evaluated by several bioassays. Chemosphere.

[79] Gabrielson J., Kühn I., Colque-Navarro P., Hart M., Iversen A., McKenzie D., Möllby R. (2003). Microplate-based microbial assay for risk assessment and (eco)toxic fingerprinting of chemicals. Anal. Chim. Acta.

[80] Gabrielson J. (2004). Assessing the Toxic Impact of Chemicals Using Bacteria.

[81] Paskuliakova A., McGowan T., Tonry S., Touzet N. (2018). Phycoremediation of landfill leachate with the chlorophyte *Chlamydomonas* sp. SW15aRL and evaluation of toxicity pre and post treatment. Ecotoxicol. Environ. Saf..

[82] Wadhia K., Dando T., Clive Thompson K. (2007). Intra-laboratory evaluation of Microbial Assay for Risk Assessment (MARA) for potential application in the implementation of the Water Framework Directive (WFD). J. Environ. Monit..

[83] Kamber M., Fluckiger-Isler S., Engelhardt G., Jaeckh R., Zeiger E. (2009). Comparison of the Ames II and traditional Ames test responses with respect to mutagenicity, strain specificities, need for metabolism and correlation with rodent carcinogenicity. Mutagenesis.

[84] Vijay U., Gupta S., Mathur P., Suravajhala P., Bhatnagar P. (2018). Microbial mutagenicity assay: Ames Test. Biol. Protoc..

[85] Wang D., Lin J., Lin J., Wang W., Li S. (2019). Biodegradation of petroleum hydrocarbons by *Bacillus subtilis* BL-27, a strain with weak hydrophobicity. Molecules.

[86] Wrenn B.A., Venosa A.D. (1996). Selective enumeration of aromatic and aliphatic hydrocarbon degrading bacteria by a most-probable-number procedure. Can. J. Microbiol..

[87] Malińska K. (2016). Application of a modified OxiTop^®^ respirometer for laboratory composting studies. Arch. Environ. Prot..

[88] Steliga T., Wojtowicz K. (2019). Wykorzystanie testów respirometrycznych do oceny efektywności biodegradacji osadów z instalacji kopalnianych. Naft. Gaz.

[89] Steliga T., Uliasz M. (2014). Spent drilling muds management and natural environment protection. Gospod. Surowcami Min..

[90] Tian L., Huang D., Shi Y., Han F., Wang Y., Ye H., Tang Y., Yu H. (2019). Method for the analysis of 7 indictor polychlorinated biphenyls (PCBs) and 13 organochlorine pesticide residues in sediment by gas chromatography (GC). IOP Conf. Ser. Earth Environ. Sci..

[91] Wojtowicz K., Jakubowicz P. (2019). Opracowanie metodyki oznaczania polichlorowanych bifenyli w próbkach gleb. Naft. Gaz.

[92] Chaîneau C.H., Yepremian C., Vidalie J.F., Ducreux J., Ballerini D. (2003). Bioremediation of a crude oil-polluted soil: Biodegradation, leaching and toxicity assessments. Water Air Soil Pollut..

