# Supplementary material for: Assessment of Biodegradation Efficiency of Polychlorinated Biphenyls (PCBs) and Petroleum Hydrocarbons (TPH) in Soil Using Three Individual Bacterial Strains and Their Mixed Culture
Source: Molecules. 2020 Feb 6;25(3):709. doi: 10.3390/molecules25030709 (PMC7036857; doi:10.3390/molecules25030709)
Supplement: Supplementary file 1 [file molecules-25-00709-s001.zip › molecules-697392-sup-proofed-2/Certificate of Analysis-Soil A.pdf]

Certificate of Analysis

Certified Reference Material

PCB Congeners in Soil

Product ID **SQC068-50G**
Lot LRAB5372
Expiration Date December 31, 2021
Storage Conditions Store at room temperature

Analyte	*Units*	*Certified Value* [1,4]	***k*** [5]	*Standard Deviation* [2]	*Acceptance Interval* [3]
PCBs, total	ug/Kg	13,100 ± 459	2.00	1,965	7,205 to 18,995
2,4,4'-Trichlorobiphenyl (PCB 28)<br>Trichlorobiphenyl, 2,4,4'-	ug/Kg	494 ± 17.3	2.00	74.1	272 to 716
2,2',5,5'-Tetrachlorobiphenyl (PCB 52)<br>Tetrachlorobiphenyl, 2,2',5,5'-	ug/Kg	607 ± 21.2	2.00	91.1	334 to 880
3,3',4,4'-Tetrachlorobiphenyl (PCB 77)<br>Tetrachlorobiphenyl, 3,3',4,4'-	ug/Kg	635 ± 22.2	2.00	95.3	349 to 921
3,4,4',5-Tetrachlorobiphenyl (PCB 81)<br>Tetrachlorobiphenyl, 3,4,4',5-	ug/Kg	784 ± 27.4	2.00	118	431 to 1,137
2,2',4,5,5'-Pentachlorobiphenyl (PCB 101)<br>Pentachlorobiphenyl, 2,2',4,5,5'-	ug/Kg	803 ± 28.1	2.00	120	442 to 1,164
2,3,3',4,4'-Pentachlorobiphenyl (PCB 105)<br>Pentachlorobiphenyl, 2,3,3',4,4'-	ug/Kg	733 ± 25.7	2.00	110	403 to 1,063
2,3',4,4',5-Pentachlorobiphenyl (PCB 118)<br>Pentachlorobiphenyl, 2,3',4,4',5-	ug/Kg	548 ± 19.2	2.00	82.2	301 to 795
2,3',4,4',5'-Pentachlorobiphenyl (PCB 123)<br>Pentachlorobiphenyl, 2,3',4,4',5'-	ug/Kg	917 ± 32.1	2.00	138	504 to 1,330
2,3,4,4',5-Pentachlorobiphenyl (PCB 114)<br>Pentachlorobiphenyl, 2,3,4,4',5-	ug/Kg	577 ± 20.2	2.00	86.6	317 to 837
3,3',4,4',5-Pentachlorobiphenyl (PCB 126)<br>Pentachlorobiphenyl, 3,3',4,4',5-	ug/Kg	1,260 ± 44.1	2.00	189	693 to 1,827
2,2',3,4,4',5'-Hexachlorobiphenyl (PCB 138)<br>Hexachlorobiphenyl, 2,2',3,4,4',5'-	ug/Kg	608 ± 21.3	2.00	91.2	334 to 882
2,2',4,4',5,5'-Hexachlorobiphenyl (PCB 153)<br>Hexachlorobiphenyl, 2,2',4,4',5,5'-	ug/Kg	571 ± 20.0	2.00	85.7	314 to 828
2,3,3',4,4',5'-Hexachlorobiphenyl (PCB 157)<br>Hexachlorobiphenyl, 2,3,3',4,4',5'-	ug/Kg	535 ± 18.7	2.00	80.3	294 to 776
2,3,3',4,4',5-Hexachlorobiphenyl (PCB 156)<br>Hexachlorobiphenyl, 2,3,3',4,4',5-	ug/Kg	757 ± 26.5	2.00	114	416 to 1,098
2,3',4,4',5,5'-Hexachlorobiphenyl (PCB 167)<br>Hexachlorobiphenyl, 2,3',4,4',5,5'-	ug/Kg	635 ± 22.2	2.00	95.3	349 to 921
3,3',4,4',5,5'-Hexachlorobiphenyl (PCB 169)<br>Hexachlorobiphenyl, 3,3',4,4',5,5'-	ug/Kg	767 ± 26.8	2.00	115	422 to 1,112
2,2',3,4,4',5,5'-Heptachlorobiphenyl (PCB 180)<br>Heptachlorobiphenyl, 2,2',3,4,4',5,5'-	ug/Kg	1,160 ± 40.6	2.00	174	638 to 1,682
2,3,3',4,4',5,5'-Heptachlorobiphenyl (PCB 189)<br>Heptachlorobiphenyl, 2,3,3',4,4',5,5'-	ug/Kg	520 ± 18.2	2.00	78.0	286 to 754
PCB (156)+(157)	ug/Kg	1,290 ± 45.2	2.00	194	710 to 1,871
PCB (20)+(28)	ug/Kg	494 ± 17.3	2.00	74.1	272 to 716
PCB (90)+(101)+(113)	ug/Kg	803 ± 28.1	2.00	120	442 to 1,164

ANAB ACCREDITED REFERENCE MATERIAL PRODUCER

ANAB ACCREDITED TESTING LABORATORY

SIGMA-ALDRICH
2931 Soldier Springs Rd. Laramie, Wyoming 82070 USA
307-742-5452
rtctechgroup@sial.com www.sigma-aldrich.com

PCB (129)+(138)+(163)	ug/Kg	608 ± 21.3	2.00	91.2	334 to 882
PCB (153)+(168)	ug/Kg	571 ± 20.0	2.00	85.7	314 to 828
PCB (180)+(193)	ug/Kg	1,160 ± 40.6	2.00	174	638 to 1,682

Sample Information

DESCRIPTION

The sample size provided is 50 g of soil.
The soil has been sterilized to minimize degradation of the sample.
The sample has been sized to 100 mesh.
The sample has been intentionally prepared with an apparent headspace.

PREPARATION INSTRUCTIONS

Mix Prior to use.
Recommended minimum sampling size is 1 gram.

STORAGE

The sample should be stored at Room Temperature. It has been determined to be stable for the duration of the expiration date.
The shelf life of the product was determined by historic stability of similar CRM's. The expiration date may be extended based on stock and popularity upon successful stability testing by a 17025 accredited laboratory.
Stability and shelf life after opening must be determined by the user, taking into account sampling frequency/volume and all local conditions.

---

1 Certified value - based on the robust mean of round robin, interlaboratory study and analytically verified by RTC with associated uncertainties from the preparation and analytical procedures.
2 The standard deviation is the robust statistical standard deviation from the round robin interlaboratory study.
3 Acceptance limits are based on Interlaboratory Study Results. These ranges are recommendations only.
4 Ucrm - Uncertainty values in this document are expressed as Expanded Uncertainty (Ucrm) corresponding to the 95% confidence interval. Ucrm is derived from the combined standard uncertainty multiplied by the coverage factor k, which is obtained from a *t*-distribution and degrees of freedom. The components of combined standard uncertainty include the uncertainties due to characterization, homogeneity, long term stability, and short term stability (transport). The components due to stability are generally considered to be negligible unless otherwise indicated by stability studies. The mathematical representation of the Ucrm calculation is as follows:

$$u_{CRM} = \sqrt{u_{char}^2 + u_{\text{hom }ogeneity}^2 + u_{stability}^2}$$

5 *k*: Coverage factor derived from a t-distribution table, based on the degrees of freedom of the data set. **Confidence interval = 95%**

Traceability: The standard was manufactured under an ISO/IEC 17025:2005 certified quality system. The balance used to weigh raw materials is accurate to +/- 0.0001g and calibrated regularly using mass standards traceable to NIST. All dilutions were preformed gravimetrically. Additionally, individual analytes are traceable to NIST SRMs where available and specified above.

Homogeneity: Homogeneity was assessed in accordance with ISO Guide 35. Completed units were sampled using a random stratified sampling protocol. The results of chemical analysis were then compared using a one-way analysis of variance approach as described by TNI EL-V3-2009 Appendix A.2. See Instructions for minimum sub-sample size.

THIS PRODUCT WAS DESIGNED, PRODUCED AND VERIFIED FOR ACCURACY AND STABILITY IN ACCORDANCE WITH ISO/IEC 17025:2005 (ANAB Cert AT-1467) and ISO GUIDE 34:2009 (ANAB Cert AR-1470).

Robert O'Brien - QC Supervisor

Mark Pooler - QA Supervisor

Certification Date July 24, 2017
Version 622-7242017
