# Supplementary material for: Assessment of Biodegradation Efficiency of Polychlorinated Biphenyls (PCBs) and Petroleum Hydrocarbons (TPH) in Soil Using Three Individual Bacterial Strains and Their Mixed Culture
Source: Molecules. 2020 Feb 6;25(3):709. doi: 10.3390/molecules25030709 (PMC7036857; doi:10.3390/molecules25030709)
Supplement: Supplementary file 1 [file molecules-25-00709-s001.zip › molecules-697392-sup-proofed-2/Supplementary materials.pdf]

molecules

MDPI

Suplementary materials

Assessment of biodegradation efficiency of polychlorinated biphenyls (PCBs) and petroleum hydrocarbons (TPH) in soil using three individual bacterial strains and their mixed culture

Teresa Steliga[1], Katarzyna Wojtowicz[1], Piotr Kapusta[2] and Joanna Brzeszcz[2]

[1] Department of Production Technology of Reservoir Fluids, Oil and Gas Institute – National Research Institute, 31-503 Krakow, ul. Lubicz 25 A, Poland

[2] Department of Microbiology, Oil and Gas Institute-National Research Institute, 31-503 Krakow, ul. Lubicz 25A, Poland

* Correspondence: teresa.steliga@inig.pl

Table 1. Comparison of PCB content in sterile soil samples A and C after inoculation with *Mycolicibacterium frederiksbergense* IN53, *Rhodocococcus erythropolis* IN129, *Rhodococcus* sp. IN306 and mixed culture M1 over 30 days. Measurement repetition number n=8–10, <0.05.

	Soil A (PCB µg/kg s.m. contaminated sterile soil)				
	Soil control	IN53	IN129	IN306	M1
PCBs	13,100 ± 915	8,391 ± 421	7,391 ± 402	5,971 ± 354	7,050 ± 371
TriCB	494 ± 28.5	202 ±15.9	162 ±11.5	123 ±9.1	134 ±10.3
TetraCBs	2,026 ± 94.5	962 ± 44.4	820 ± 41.5	678 ± 39.4	740 ± 41.2
PentaCs	4,838 ± 185.1	2,862 ± 112.2	2,814 ± 127.9	2,172 ± 99.6	2,618 ± 104.8
HexaCBs	3,873 ± 121.2	2,967 ± 105.1	2,392 ± 92.1	1,962 ± 71.6	2,349 ±88.2
HeptCBs	1,680 ± 82.1	1,398 ± 71.2	1,202 ± 65.9	1,034 ± 51.6	1,109 ± 64.9
	Soil C (PCB µg/kg s.m. contaminated sterile soil)				
	Soil control	IN53	IN129	IN306	M1
PCBs	13,100 ± 915	9,243 ± 358	8,001 ± 454	6,853 ± 387	6,309 ± 365
TriCB	494 ±28.5	241 ± 19.5	192 ±15.8	151 ±11.9	146 ±10.9
TetraCBs	2,026 ± 94.5	1,168 ± 55.3	934 ± 45.4	764 ± 38.9	761 ± 38.5
PentaCs	4,838 ± 185.1	3,250 ± 132.8	3,055 ± 145.6	2,525 ± 93.3	2,277 ±99.2
HexaCBs	3,873 ± 121.2	3,137 ± 111.4	2,562 ± 89.5	2,233 ± 84.1	2,060 ± 82.6
HeptCBs	1,680 ± 82.1	1,449 ± 88.2	1,256 ± 51.3	1,184 ± 53.5	1,059 ± 51.9

Table S2. Comparison of TPH, unidentified hydrocarons, alkanes (ΣnC_{10}–nC_{22}, ΣnC_{23} – nC_{40} content in sterile soil samples B and C after by *Mycolicibacterium frederiksbergense* IN53, *Rhodococcus erythropolis* IN129, *Rhodococcus* sp. IN306 and mixed culture M1 after the 30 day biodegradation process. Measurement repetition number n=8–10, <0.05.

	Soil B (TPH mg/kg s.m. contaminated sterile soil)				
	Soil control	IN53	IN129	IN306	M1
TPH	12,515 ± 787	7,863 ± 398	8,951 ± 451	9,396 ± 487	8.309 ± 402
Unident. hydrocarb.	5,687±258	3,825± 198	4,115 ± 214	4,223 ± 247	3,990 ± 214
ratio nC_{17}/pristane	0.818 ± 0.04	0.473 ± 0.02	0.536 ± 0.02	0.594 ± 0.02	0.522 ± 0.02
ratio nC_{18}/phytane	1.657 ± 0.09	0.985 ± 0.07	1.154 ± 0.08	1.232 ±0.08	1.029 ± 0.08
Σ nC_{10} – nC_{22}	4,058 ± 187	2,154 ± 135	2.589 ±128	2.788 ±112	2,292 ± 108
Σ nC_{23} – nC_{40}	1,956 ± 178	1,183 ± 99	1,546 ± 139	1,658 ± 147	1,334± 129
	Soil C (TPH mg/kg s.m. contaminated sterile soil)				
	Soil control	IN53	IN129	IN306	M1
TPH	12,365 ±769	8,282 ± 411	9,426 ± 499	9,838 ± 529	8,085 ±396

Unident. hydrocarb.	5,536 ± 247	3,909 ± 196	4,345 ± 247	4,541 ± 274	3,874 ± 228
ratio nC_{17}/pristane	0.819 ± 0.04	0.541± 0.02	0.489 ± 0.02	0.601 ± 0.02	0.495 ± 0.02
Ratio nC_{18}/phytane	1.657 ±0.09	1.167 ± 0.08	0.996 ± 0.07	1.266 ± 0.08	1.009 ± 0.07
Σ nC_{10}-nC_{22}	4,076 ± 288	2,336 ± 145	2,732 ± 165	2,876 ± 128	2,235 ±114
Σ nC_{23}-nC_{40}	1,956 ± 198	1,321 ± 117	1,614 ± 142	1,686 ± 145	1,264 ± 109

Table S3. Coefficients of first-order mathematical model describing PCB and TPH pollutants group biodegradation in non-sterile soil D (ex-situ prism method). Measurement repetition number n=7–10, <0.05.

PCB pollutants group	k [day^{-1}]	$(C/C_{PCB209})_0$	Determination coefficient (r^2)
PCBs	0.0109 ± 0.0009	16,102 ± 80.5	0.9928
TriCB	0.0170 ± 0.0012	240.6 ± 12.0	0.9883
TetraCBs	0.0110 ± 0.0008	545.8 ± 27.3	0.9897
PentaCBs	0.0095 ± 0.0007	593.1 ± 29.7	0.9908
HexaCBs	0.0082 ± 0.0006	216.1 ± 10.8	0.9853
HeptCB	0.0050 ± 0.0003	21.0 ± 1.1	0.9741
Petroleum pollutants group	k [day^{-1}]	$(C/C_H)_0$	Determination coefficient (r^2)
TPH	0.0070 ± 0.0004	4,087.1 ± 204.4	0.9841
Σ nC_{11} – nC_{22}	0.0130 ± 0.0009	1,892.2 ± 94.6	09941
Σ nC_{23} – nC_{40}	0.0049 ± 0.0003	867.5 ± 43.4	0.9976

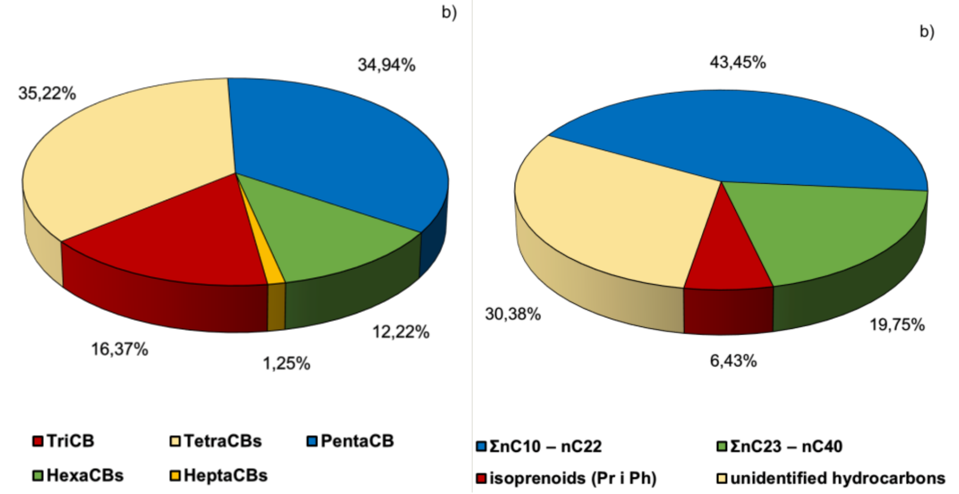

Figure S1: Percentage share of identified pollutants in the sample (Soil D) comprising: a) PCBs, b) TPH

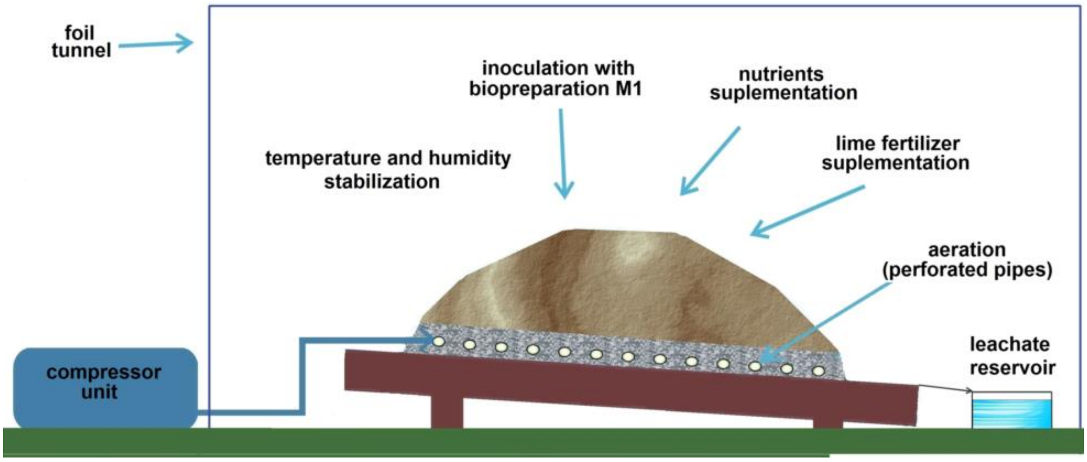

Figure S2: Scheme of the test stand for conducting the process of biodegradation of pollutants (PCBs and TPH) in soil D under semi-technical conditions (*ex situ* prism method), Certificate of Analysis Soil A, Ecotoxicological analyses –description of the methodology

Ecotoxicological analyses –description of the methodology

Ostracodtoxkit(F)™ (MicroBioTest Inc. Belgium

Ostracodtoxkit(F) test belongs to direct contact tests of chronic toxicity estimation with the use of crustaceans *Heterocypris incongruens*. The direct contact test is done with the application of young bottom crustaceans (*Heterocypris incongruens*), which hatched from cysts during 52 hours (according to the producer's procedure). The test is carried out on 6-hole polystyrene microplates. A standardised nutrient is an algae suspension, which is applied to the suspension in portions of 2 ml. Portions of 10 ostracods are added to each cell of the microplate. 0.5 g of a control soil is applied to the row A, whereas 0.5 g of the soil sample is added to the consecutive rows. Incubation lasts for 6 days in a temperature of 25°C. Results reading consists of estimation of life microforms amount in each hole of the microplate and measurement of their length. Growth inhibition of H. incongruens was calculated as

$$GI = 100 - (\frac{A}{B} * 100)$$

where: A – ostracods length in tested soil, B – ostracods length in reference soil

Microtox® Solid Phase Test (SDI, USA)

Microtox Test created in the USA in 1979 as the first bioindication test, combines typical bioindication and analytic precision. Luminescence bacteria *Vibro fisch*eri, which uses about 10% of its metabolism in order to emit light, was used as a bioindicator. In the presence of toxical substances, there is decrease in luminescence and increase in general toxicity of a sample. In an electron transportation system of the bacteria, lucipherasa enzyme (alcane oxygenaze) catalyses oxidation of a reduced substrate (reduced flavine mononucleotide, riboflavin phosphate or flavine adenin dinucleotide) and during this process luminescence, which can be measured with a photometer, takes place. The obtained substrates of this reaction are oxygen and long-chain aldehyde. In the presence of substances that have negative influence on cell metabolism, the decrease in luminescence of bacteria is immediate[1].

Test Microtox®, produced by SDI Company (USA), enables a direct contact of luminescence bacteria *Vibro fischeri* with a wastewater sample, which leads not only to determination of substances dissolved in water, but also to recognition of lipophilicity systems and poorly dissolved systems in water. Lyophilised bacteria *Vibro fischeri* can be stored in a period of a year in temperature of -20°C and then used for testing immediately after suspending in deionised water. A tests with serial dilutions of the wastewater were done according to a standard procedure and results were obtain in Delta Tox analyser. In this analysis, the concentration of wastewater, which reduces the luminescence by 50%, was determined (half

maximal effective concentration, EC_{50}). In this presentation a lower of EC_{50} denotes a higher toxicity. To simplify this interpretation, EC_{50}' values were converted to Toxicity Units (TU) in which a higher value denotes a higher toxicity according to the following formula.

$$TU = \frac{1}{EC_{50}} * 100$$

Phytotoxkit™ test (MicroBioTests Inc., Belgium)

Chronic toxicity assessment test *Phytotoxkit™* is based on the evaluation of germination and early growth of plants (root elongation inhibition measurement. Three types of plants selected according to germination rate and root growth rate are used in the test, which allows making a complete determination in 3 days of incubation: monocotyledonous - sorghum *(Sorghum saccharatum),* and dicotyledonous - cress *(Lepidium sativum)* and white mustard *(Sinapis alba).* The determination process was carried out using three repetitions for each test plant. The tests were performed on transparent polystyrene test plates. *Incubation conditions:* temperature T = 25°C in darkness, incubation time t = 72 h. *Test reaction:* inhibition of germination and early root growth.

MARA environmental risk test

Evaluation of the data produced with the MARA is assessed by examining the growth inhibitory effect on the pellet formation at each sample dilution. The assessment can be made for each individual species or by looking at the mean software computed value of all the constituent species. Using the latter the toxicity of a sample can be expressed with reference to a threshold value or interval assigned for toxicity classification. For example, an undiluted sample exhibiting an overall inhibitory effect (across 11 species) of o20% is considered to be non-toxic. This is an effective means of obtaining a rapid indication (or assessment) of the results.

In order to provide a comprehensive and optimal assessment utilising the significant feature of the MARA as a multi species test, a determination referred to as the Microbial Toxic Concentration (MTC) is computed. The MTC value is determined as follows

$$MTC = c_{min} x\ d^{(Ptot/P0)-1}$$

where c_{min}=lowest concentration in the gradient, P_0=pellet size in the control, d= dilution factor and P_{tot}= six pellet sizes down the concentration gradient.

The MTC is a means of computing a value equivalent to the EC_{50} determination. The computation uses all available points above and below the growth curve in the 0–100% inhibition range[3].

As the MARA plate generates 11 independent values from 11 microbial strains, a test result can be presented either as the mean inhibition value or as the minimum inhibition value if a single numerical value is required. The mean value is probably a more "realistic" value, a common criticism against existing bacterial assays is that the result from one strain only is not representative. On the other hand, the minimum value is a valuable result as it shows at which concentration level the most susceptible species may be affected. However, most of the information gained in a multi-species test is lost if a single value is used for evaluation instead of the whole toxic fingerprint[4].

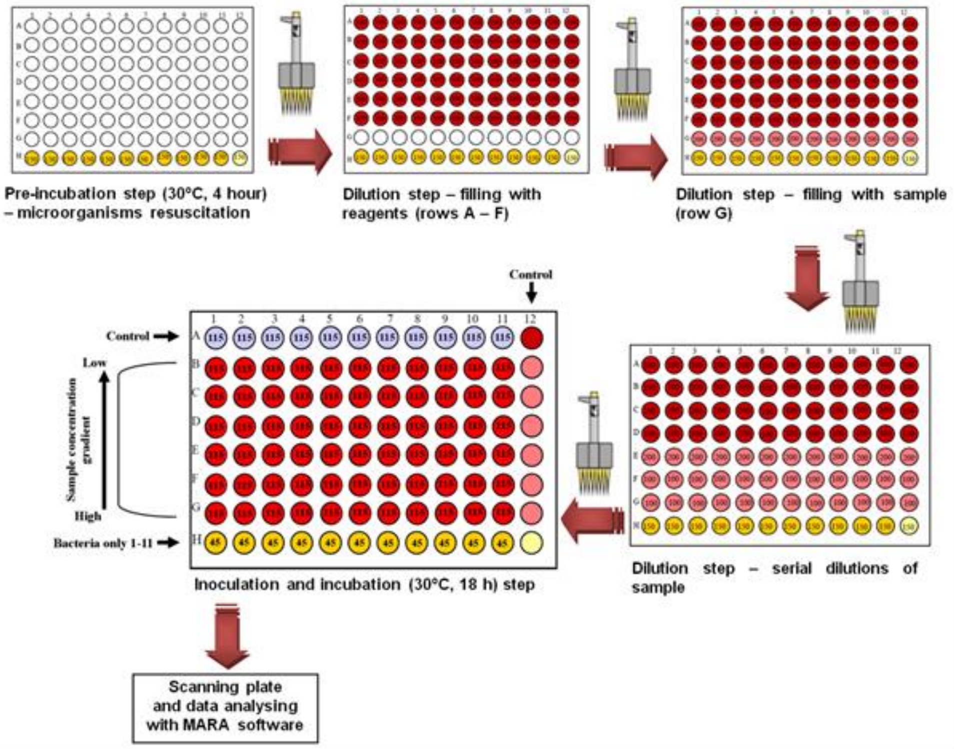

Figure S3. Scheme of MARA test conduct

AMES (Muta-ChromoPlate™ Kit (EBPI, Canada).

Numerous petroleum contaminants and metabolites created in biodegradation of pollutants include carcinogenic compounds. In order to estimate potentially mutagenic properties of the wastewater, Ames Test was applied. It enables determination of reverse mutations from histidine auxotrophy to prototrophy in mutants, which are test strains of *Salmonella typhimurium*. The test strains of the bacteria can be characterised by various types of mutation in genes, which are responsible for histidine synthesis. Therefore, the presence of histidine in a substratum is required in order to provide the growth of the strains. Generally, TA98 and TA100 strains are used[5]. They include proper mutations consisting of so-called reading frame displacement, pair base substitution and two mutagenes (2-nitro-fluoren and sodium azide). The test strains are mutants, which are unable to histidine synthesis. A

mutagenous substance causes reversion in mutation and histidine synthesis can take place, which results in appearance of revertants colonies in the histidine-free basis.

In order to lead Ames Test, a high-tech microplate AMES MPF test was applied. It can be used for detection of genotoxic activity of water contaminated with petroleum substances and metabolites, which are the effects of biodegradation. Delivered microforms of T-98 and T-100 *Salmonella typhimurium* undergo strict quality control (genotype and phenotype). The test results in colour reaction, where yellow means reverse mutation, whereas scarlet – the lack of mutation.

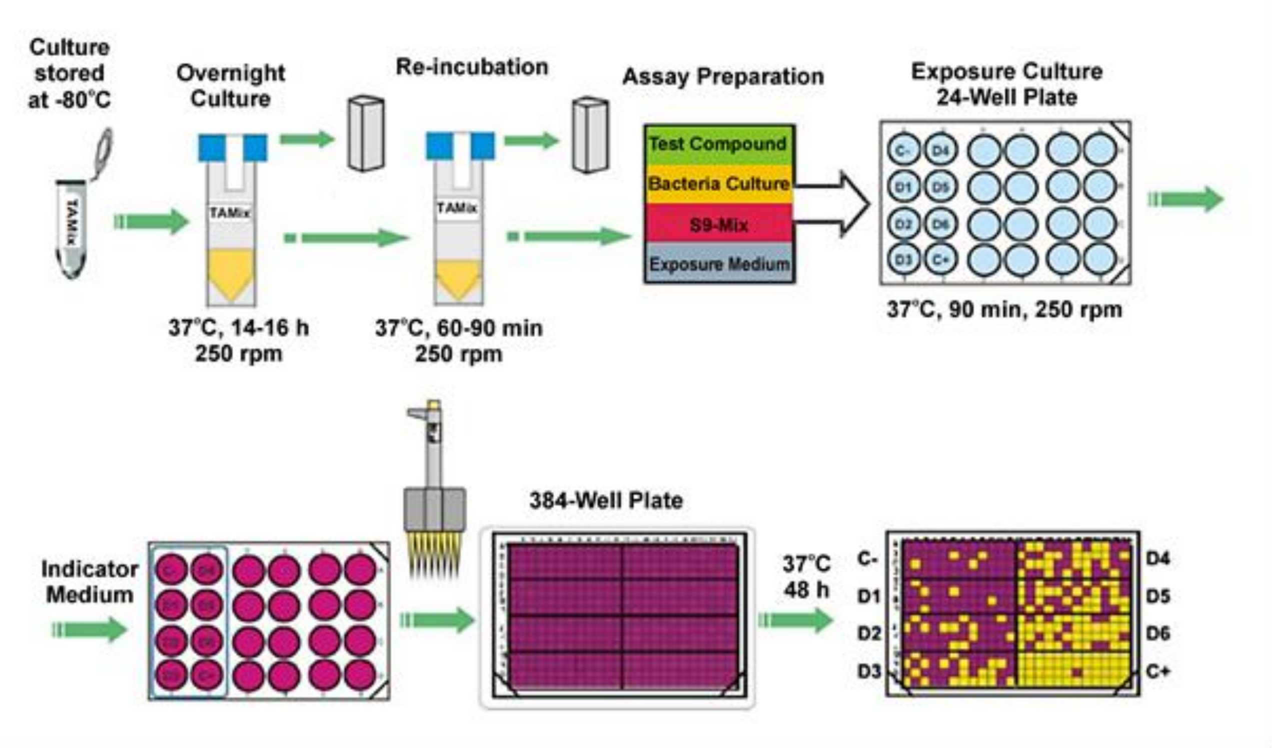

Figure S4. Scheme of Ames Test conduct

Petroleum contaminants from the samples were extracted with the use of dichloromethane, which was then vaporised and the obtained deposit was dissolved in dimethylosulfoxide (DMSO)[6]. As an activator, S9 microsomal fraction from a rat liver was used. The role of the activator is to transform the tested substance, which leads to disclosure of its potentially mutagenous properties.
